# Bidirectional regulation of gut microbiota and young ruminant host: implications for intestinal development and mucosal immunity

**DOI:** 10.1186/s40104-026-01441-8

**Published:** 2026-06-18

**Authors:** Mulugeta Walelegne, Ma Junnan, Yuan Hao, Xiaoran Feng, Hunegnaw Abebe, Ruochen Yang, Luxin Kong, Yan Tu

**Affiliations:** 1 Beijing Key Laboratory for Dairy Cow Nutrition, Institute of Feed Research, Chinese Academy of Agricultural Sciences, Beijing, 100081 China; 2 Ethiopian Institute of Agricultural Research, Holetta Agricultural Research Center, P.O.Box 31, Holetta, Ethiopia; 3 College of Agriculture, Department of Animal Sciences, Wollo University, P.O. Box 1145, Dessie, Ethiopia

**Keywords:** Gut microbiota, Host, Intestinal development, Mucosal immunity, Young ruminants

## Abstract

As global demand for milk and meat increases, young ruminants need rapid gut development and sufficient mucosal immunity immediately after birth. However, their growing intestines face oxidative and microbial challenges that can harm long-term performance. This review systematically summarizes recent advances in how host genetics, early nutrition, and emerging microbiota interact to influence intestinal development and mucosal immunity under standard rearing conditions. We explore how the host regulates the gut microbiota, the microbiota’s role in maintaining gut integrity, barrier function, development, intestinal cell health, mucosal immunity, and how diet modulates gut microbiota composition in young ruminants. Microbial signals promote increased villus length and better epithelial functions. Conversely, dysbiosis delays gut closure, weakens barrier integrity, and skews immunity toward pro-inflammatory responses. Feeding strategies such as colostrum timing, milk replacers, and the addition of starter fiber and probiotics can alter microbial communities within days. Nonetheless, challenges remain in standardizing neonatal feeding practices, identifying microbe metabolite indicators of gut health, and integrating precision feeding technologies. By mapping the three-way interaction among host, microbiota, and diet, this review offers a blueprint for neonatal ruminant feeding that enhances both animal welfare and the productivity of future ruminant systems. This review also provides a novel mechanistic integration of host genetics, gut microbiota succession, and dietary interventions in young ruminants, filling the gap of fragmented multi-omics analyses in existing literature and offering targeted insights for optimizing gastrointestinal development and production efficiency.

## Introduction

The gut microbiota of young ruminants is a dynamic and multifaceted community that plays a key role in intestinal and mucosal immune development [1, 2]. It is a rapidly proliferating community that is established immediately post-birth, and early gut colonization significantly contributes to the development of gut-associated lymphoid tissue (GALT), differentiation of immune cells, and development of immune tolerance [2, 3]. Transcriptomic assessment has revealed that the intestinal mucosal immune system is strongly remodeled in the first week of life, which is due to microbial colonization and microRNAs [4]. Simultaneously, the host provides a nutritional niche and immune profiles that define the gut microbiota structure and function, ensuring a symbiotic relationship [5]. The ontogeny of young ruminants has the effect of regulating the colonization of microbes via immunological as well as genetic mechanisms [6]. Early microbial assemblages are formed at parturition by contact with the skin and milk of the dam [7]. These initial exposures and host-derived chemo signaling determine the taxa capable of colonizing the gut. Colonization mechanisms are also under genetic control by pattern-recognition receptors, such as NOD-like and Toll-like receptors, which coordinate responses to manipulation of the microbial community [8, 9]. The interplay between the microbiota and the immune system plays a major role in mucosal defense and maturation [3]. The selective production of antimicrobial peptides, including defensins and cathelicidins, targets pathogenic bacteria while sparing beneficial bacteria to maintain host-mediated balance [10]. Moreover, immune proteins such as secretory IgA enable bacterial adhesion, prevent adhesion to the intestinal mucosa, and promote tolerance to intestinal microorganisms [11].

The host genetics also control the microbial environment through the production of physicochemical parameters. For example, gradients of oxygen tension, pH, and bile acids, traceable up and down, resonate in the niches of gastrointestinal colonization by microbes [12, 13]. These conditions shift owing to the growth of the calf, especially during weaning, when dietary changes alter intestinal activities [14]. This is promoted by hormonal and dietary nutrients that help to form papillae, increase vascular flow, and alter gut motility and digestive function [15]. These changes help enable the uptake of microbial fermentation end-products, such as short-chain fatty acids (SCFAs), a form of signaling molecules, presented to the host cells [16]. It also helps to feed the gut mucosa, strengthen the epithelial barrier, and inhibit inflammation, which contributes to promoting healthy host-microbe interactions [3, 17].

However, gut microbiota imbalance in the host, dietary changes, and abrupt weaning cause dysbiosis, barrier malfunction, and increased vulnerability to intestinal injury and inflammatory diseases [1, 18, 19]. To balance this situation, the gut microbiota produces SCFAs and secondary bile acids to regulate immune cell differentiation and cytokine secretion, and the epithelial barrier significantly contributes to the regulation of innate and adaptive mucosal immunity [20–22]. Various microorganisms that produce SCFAs differ in metabolite production, for example, Firmicutes (syn. Bacillota) being the most common butyrate producers [23, 24]. These metabolites not only support anti-inflammatory activity and the development of regulatory T cells but also strengthen the mucosal and immune systems of the gastrointestinal tract [25, 26]. Microbial extracellular vesicles and metabolites also serve as mediators of interkingdom communication, regulators of host signaling pathways, and systemic immune responses [21, 27]. In addition, the immune response and surveillance establish a balance between commensal and pathogenic microbes, as the host immune system regulates microbial assemblies through the active governance of microbial signals [28, 29]. Such crosstalk occurs primarily during early life, when the immune system and gut microbiota develop simultaneously, with long-lasting effects on host physiology [30, 31].

In neonatal ruminants, nutritional strategies such as supplementation with yeast cultures, probiotics, prebiotics, and postbiotics have the potential to promote the growth of beneficial microbes, support mucosal immune development, and mitigate the negative effects of weaning stress [32, 33]. The mechanisms underlying these strategies include the initial establishment, restoration, or preservation of microbial homeostasis, the promotion of epithelial proliferation, and the enhancement of immune protection, thereby reducing the prevalence of gastrointestinal disorders and enhancing overall growth and well-being [34–36]. In this regard, the interaction between diet, microbiota, and host immunity is the most important determinant of intestinal development and resistance to disease in early life [37, 38]. The intestinal microbiota also serves as a satellite organ by facilitating digestion and protection, while also synthesizing essential homeostatic molecules involved in metabolism and the structure of the adaptive immune system [39]. Despite significant advancements in elucidating gut microbial alterations and nutritional strategies in young ruminants, current reviews have several unresolved issues: most previous studies emphasize descriptive microbial profiles rather than the mechanistic bidirectional interactions between the host and microbiota, do not differentiate species-specific responses from overarching patterns across ruminants, and lack a unified framework connecting microbial metabolites to intestinal maturation and mucosal immune development. Furthermore, the context-dependent regulatory nodes governing early life microbial succession and immune development remain poorly defined, constraining the applicability of foundational discoveries to a particular nutritional regimen. This review specifically emphasizes these constraints by incorporating mechanistic insights, multi-omics evidence, and species-specific data to analyze host-gut microbial interactions, while elucidating speculative extrapolations and evidence-based generalizations about young ruminants.

## Methodology

**Fig. 1:**
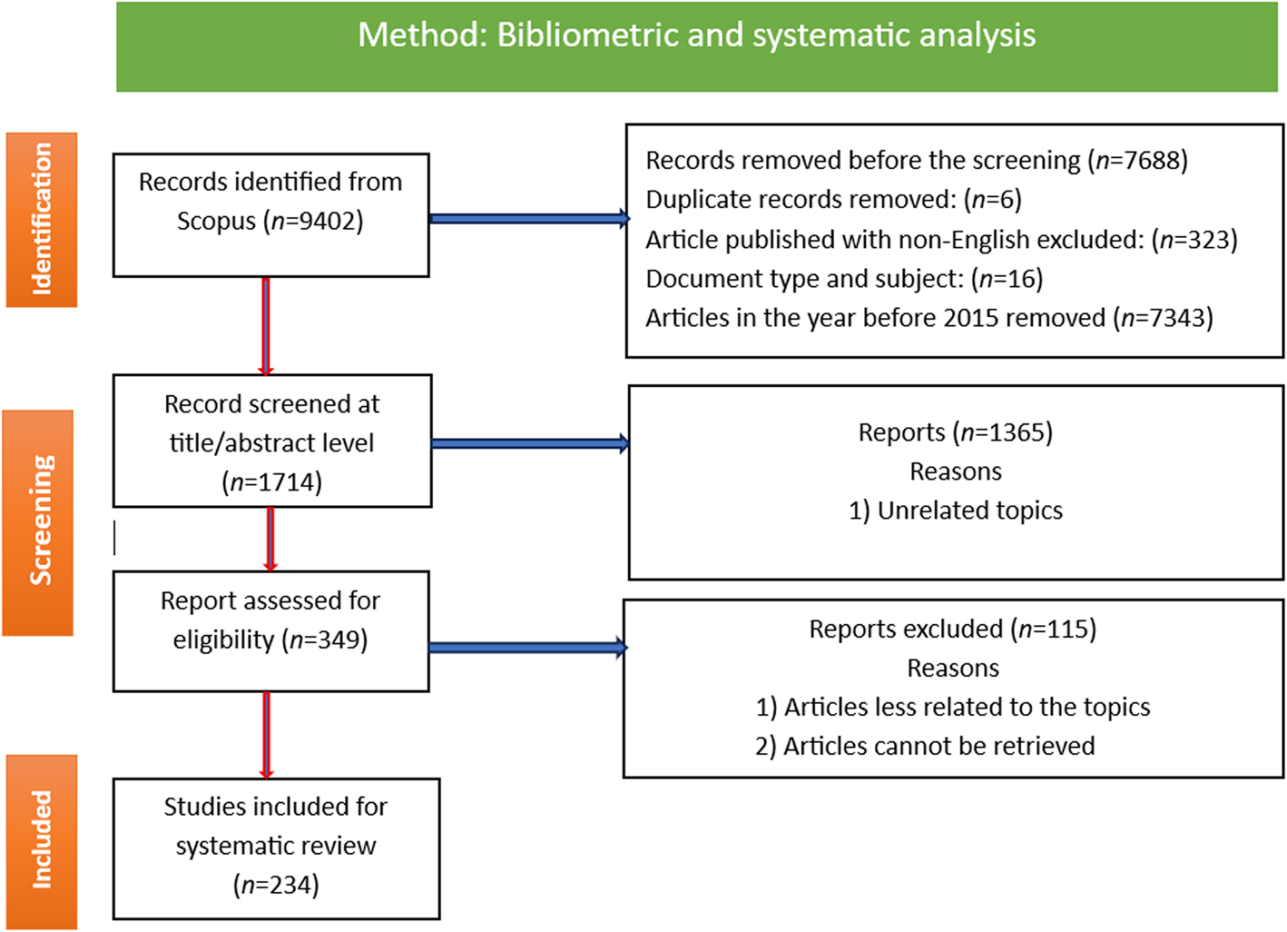
Flow chart of the study selection, inclusion, and exclusion process

This review systematically gathered, analyzed, and synthesized research on the regulation of the interaction between gut microbiota and the host in young ruminants. A literature review search was performed based on the PRISMA guideline, which suggests documentation of items in systematic reviews. Literature search was conducted using Scopus, Science Direct, PUBMED, and Web of Science, between 2015 and 2025. The search strategy was as follows: intestinal microbiome OR rumen microbiota OR crosstalk OR host-microbe interaction OR mucosal immune system OR young ruminants OR calves OR kids OR lambs, and a search of the respective keywords was conducted in the international literature database. We also applied the subject and keywords with the Boolean operators AND/OR to identify target articles, and the language was restricted to English to identify intersections. In addition, the reference lists of the selected articles were reviewed to identify additional relevant articles (Fig. 1). The inclusion criteria focused mainly on the published works related to ruminant species, including cattle, sheep, and goats, which were considered eligible for selection. Concerning age, the inclusion scope covered studies involving pre-weaned and post-weaned ruminants, as well as growing and adult individuals, provided that the age group was clearly reported in the original publication. Studies were excluded if they did not provide sufficient original quantitative or qualitative data relevant to the research objectives, including editorials, letters, commentaries, and conference abstracts without full data. For this comprehensive review, studies were excluded if they were editorials, letters, conference abstracts, or lacked complete usable data. Methodological quality and risk of bias were formally assessed for all included studies, as a core step of the systematic review to maintain rigor and ensure the reliability of synthesized evidence.

**Fig. 2:**
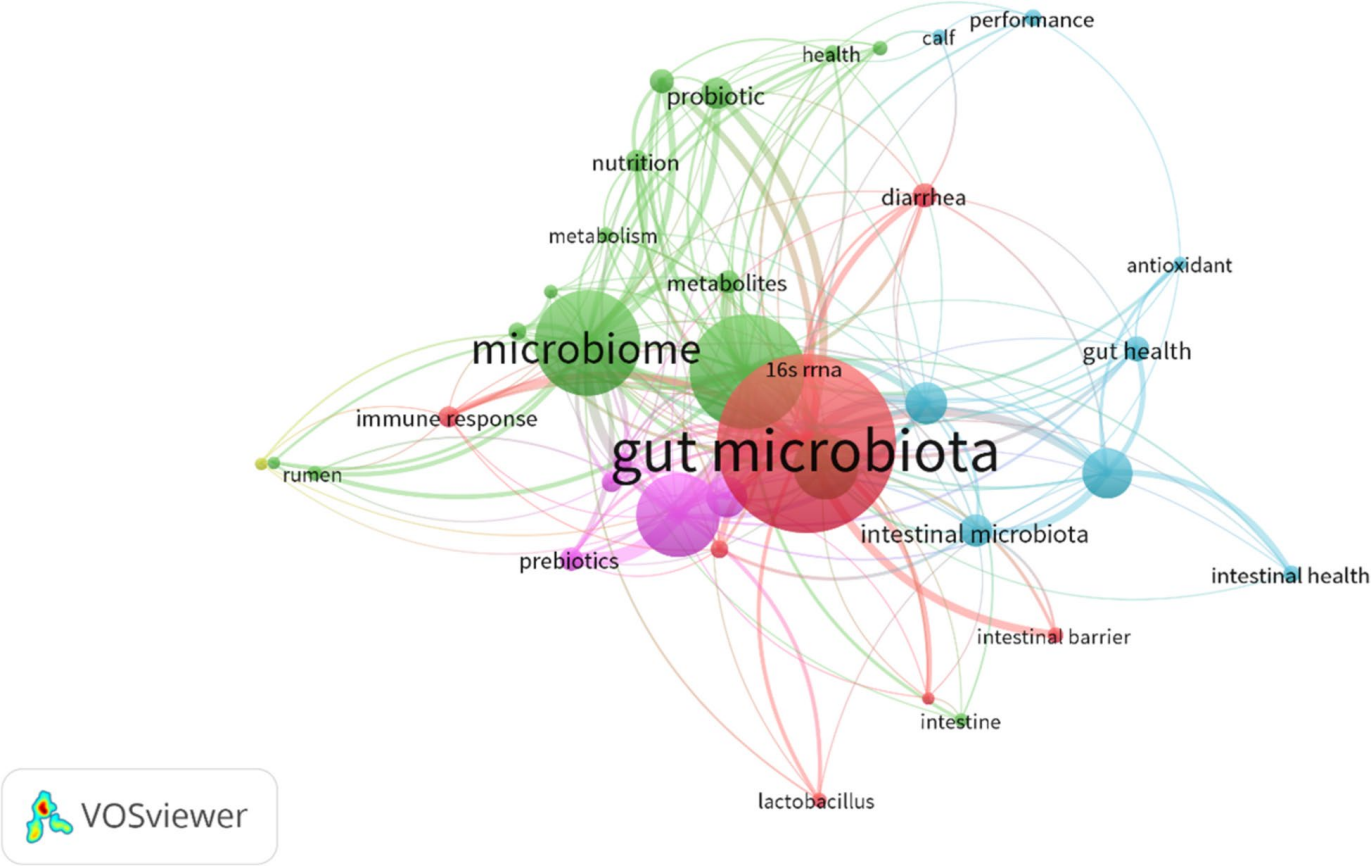
The keyword co-occurrences of gut microbiota-related research 2015–2025. The nodes refer to the frequency of documents where the keyword was quoted. The node colors refer to the cluster where the keyword belongs

Overall, 1,201 keywords were found in the recorded information, each occurring only once. The count of the top frequently occurring keywords was estimated at 46. The most common frequency was 21–56, with gut microbiota at the top (56), microbiome at the second position (53), microbiota (51), and rumen (47). Approximately 16 keywords that represented names of places (countries) were eliminated, and the remaining 73 keywords were used to conduct the analysis. These keywords were grouped into five clusters using the VOS viewer software (Fig. 2). VOSviewer analysis was used to map keyword co-occurrence and thematic networks among the included literature, supporting clear biological interpretation by revealing hidden research links, highlighting core biological themes, and mapping out the full landscape of the field, while complementing rather than replacing systematic data synthesis to deepen holistic biological understanding.

## Core functions of gut microbiota on intestinal development and health of young ruminants

### Maintenance of intestinal integrity and barrier function

The intestinal microbiota plays a key role in establishing and maintaining gut integrity and barrier function in young ruminants. The maternal nutrition and the gastrointestinal microbiome of ruminants are the primary determinants of gut shapes and the gut microbiota of the offspring [15]. Maternal microbiota and nutrition determine early exposure to microbes needed to colonize the gastrointestinal tract (GIT), such as the rumen and intestine, and establish a normal gut ecosystem [40]. However, this interpretation remains controversial, as certain identified prenatal microbial signals may represent DNA contamination rather than viable colonizers [41]. The GIT colonization, particularly by lactic acid bacteria and bifidobacteria, helps with epithelial barrier maturation through the upregulation of tight junction proteins (claudins and occludin) [42–44]. Other commensal bacteria also adjust barrier function by influencing prominent signaling pathways, such as by downregulating epithelial neuropilin-1 and hedgehog signaling [45]. Microbial-derived metabolites, including short-chain fatty acids, amino acids, and bile acids, serve as principal regulators of barrier function and influence signaling pathways that govern tight junction proteins and permeability [42, 46]. These metabolites are pivotal in the organization of the intestinal barrier when animals transition from suckling to weaning [47, 48]. Among these, butyrate preserves the function of epithelial cells, upregulates tight junction proteins such as ZO-1 and occludin, and balances physiological hypoxia to enhance barrier strength [49]. Nonetheless, existing evidence connecting microbial metabolites to intestinal barrier maturation is predominantly correlative, and direct causal linkages are not yet adequately demonstrated [50, 51]. For example, supplementation of *Clostridium butyricum* increased fecal butyrate and propionate concentrations and reduced diarrhea incidence in preweaning Holstein calves, supporting a beneficial role of short-chain fatty acids in intestinal health [50]. The underlying mechanisms also remain incompletely characterized, as much of the available evidence is still derived from in vitro systems that may not adequately reflect ruminant intestinal physiology. Accordingly, many studies reported that a stable and diversified microbiota is a key determinant for sustaining the optimal integrity of the intestinal barriers because it enhances the physical and chemical protective mechanisms to alleviate translocation of pathogens [52, 53]. This dysbiosis promotes the growth of pathogens and compromises the system by increasing gut permeability [54]. This condition is further worsened at the preweaning and weaning stages, which are marked by significant dietary and microbial community changes [55]. At this point, stress-induced inflammatory responses may change the distribution of tight junctions, make paracellular permeability higher, and help lipopolysaccharide move through the lumen, which raises the risk of enteric disease [56, 57]. For example, dietary strategies aimed at the milk-to-solid transition can alter microbial composition and restore or enhance tight junction and mucin expression, thereby decreasing disease risk [58–60]. In line with this, interventions such as probiotic supplementation have been reported to improve gut barrier function, reduce pathogen load, and enhance overall health and development [61]. Overall, the intestinal microbiota is closely related to the development of barriers; however, the exact microbial taxa, metabolites, and host pathways that are directly involved are still not well understood. Consequently, additional longitudinal, diet-controlled, and ruminant-specific mechanistic studies, supported by standardized multi-omics methodologies, are essential to elucidate the direct regulators of intestinal barrier function in young ruminants [62].

### Promotion of gut structure and development

The structure of the gut and the development of the villi depend on the composition and the function of the gut microbiota. To establish strong immune capacity and barrier function, the rumen and intestine must develop properly during the pre-weaning period. This will reduce the animal’s susceptibility to disease and ensure that it can adapt to a variety of dietary environments in later life [63, 64]. Growth performance and nutrient absorption may be permanently hampered if gut development is not optimized during this delicate stage [65, 66]. It has been reported that *Macleaya cordata* (MCE) supplemented until weaning resulted in a remarkable increase in the length of the ruminal papillae, villus height, and the ratio of villus height to crypt depth in the small intestine [67]. In another study, the development and enrichment of the structure and diversity of the mucosal microbiota was found to enhance the growth and villus formation in juvenile sika deer [61], which may provide insights into similar processes in other juvenile ruminants. Changes in the intestinal microbiome composition also enhance intestinal weight, length, and villus-crypt morphology [68].

Furthermore, the presence of certain microbial taxa, such as Lachnospiraceae and Ruminococcaceae, has been associated with improvement of villus structure and intestinal function [69, 70]. SCFAs are also capable of stimulating the proliferation and differentiation of intestinal cells, which results in the expansion of villi and improvement of mucosal turnover [71]. However, the contribution of SCFAs, particularly the effect of butyrate on gut structural development, also appears to be context dependent. Although sodium butyrate supplementation has been reported to increase ruminal papillae length and width [72], whereas other studies found no effect on papillae length when butyrate was supplied as tributyrin in calf starter [73]. These discrepancies might be due to being sometimes overridden by factors such as the animal’s age, the overall diet composition, and the dosage and form of butyrate administered. This enhancement correlates with the activation of signaling pathways, such as Wnt/β-catenin, involved in intestinal stem cell function and villus development [74]. Notably, evidence directly linking specific microbial taxa to Wnt/β-catenin signaling in ruminants remains limited, and much of the mechanistic understanding comes from non-ruminant models. In addition, caecal microbiomes have been linked to villus height and improved growth performance, highlighting the role of microbial succession in early-life development [75]. Moreover, the microbiota controls genes that regulate the epithelial barrier and angiogenesis, which determine the restructuring of villus capillary networks necessary for nutrient absorption and gut health [76]. Similarly, the regulatory interplay between nutrient metabolism, the innate immune system, and commensal microbiota helps to modulate cellular and morphological intestinal features, such as epithelial lineage regeneration and maturation, and capillary networks of the villus structures [77]. Current evidence supports a close link between the gut microbiota and intestinal structural development, but it remains unclear whether specific microbial taxa and metabolites directly regulate villus and papillae growth or accompany changes in diet and host development. Therefore, more mechanistic studies are needed to distinguish direct microbial effects from secondary nutritional and growth-related influences.

### Safeguarding of intestinal cell health and function

The intestinal microbiota maintains the health and functional integrity of intestinal epithelial cells through various regulatory mechanisms. For example, resident microbiota activate the production of regulatory immune cells, such as IL-10-producing B cells, through TLR2/MyD88/PI3K signaling pathways, which maintain both the repression of mucosal inflammation and colonic homeostasis [78]. Intestinal microbiota and epithelial cell interdependence are highly site-specific in ruminants, and local crosstalk forms organ-level functionality and physiology [79]. The mucosal barrier is composed of a single epithelial cell that physically and chemically separates the microbiota and the host immune system to maintain homeostasis [48]. Intestinal epithelial cells release a host of immunological mediators, such as chemokines and cytokines, that regulate host immunity and maintain a balanced host-microbe interaction on microbial activation [80]. The microbiota also maintains epithelial barrier health and conditions the mucosal immune system, with an immune tolerance balance by microbial metabolites and direct cellular contacts [81]. Similarly, epithelial cells perceive and react to microbial signals, controlling proliferation, differentiation, and barrier integrity [82]. Accordingly, the maintenance of gut barrier homeostasis depends on bidirectional regulation between epithelial cells, immune cells, and the microbiota, with dysregulation leading to disease and inflammation [83]. The commensal bacteria are not only effective in gut maturation and integrity but also prevent pathogens and regulate the immune responses [84]. In this situation, specialized epithelial lineages, such as Paneth and goblet cells, work together with microbiota to ensure homeostasis and barrier functions [85]. In the face of infections, epithelial cells organize innate immune-stressed responses, mucus secretion, and the release of cytokines to counter the pathogen and regulate their interaction with the microbiota [86]. Epithelial cells also coordinate the signaling of commensal microbes and structure the regulatory pathways that define the role and recruitment of immune cells [87]. However, most mechanistic evidence has been derived from in vitro cell systems or non-ruminant models, which may not fully capture the complexity of epithelial microbiota interactions in young ruminants. Generally, intestinal immune homeostasis and resistance to disease in young ruminants cannot be achieved without a dynamic interaction between gut microflora and epithelial cells [28]. Therefore, prioritizing ruminant-specific mechanistic approaches to clarify how microbial signals directly regulate intestinal cell health and function is crucial.

## Bidirectional regulation between the host and gut microbiota

### Colonization dynamics of gut microbiota in young ruminants

**Fig. 3:**
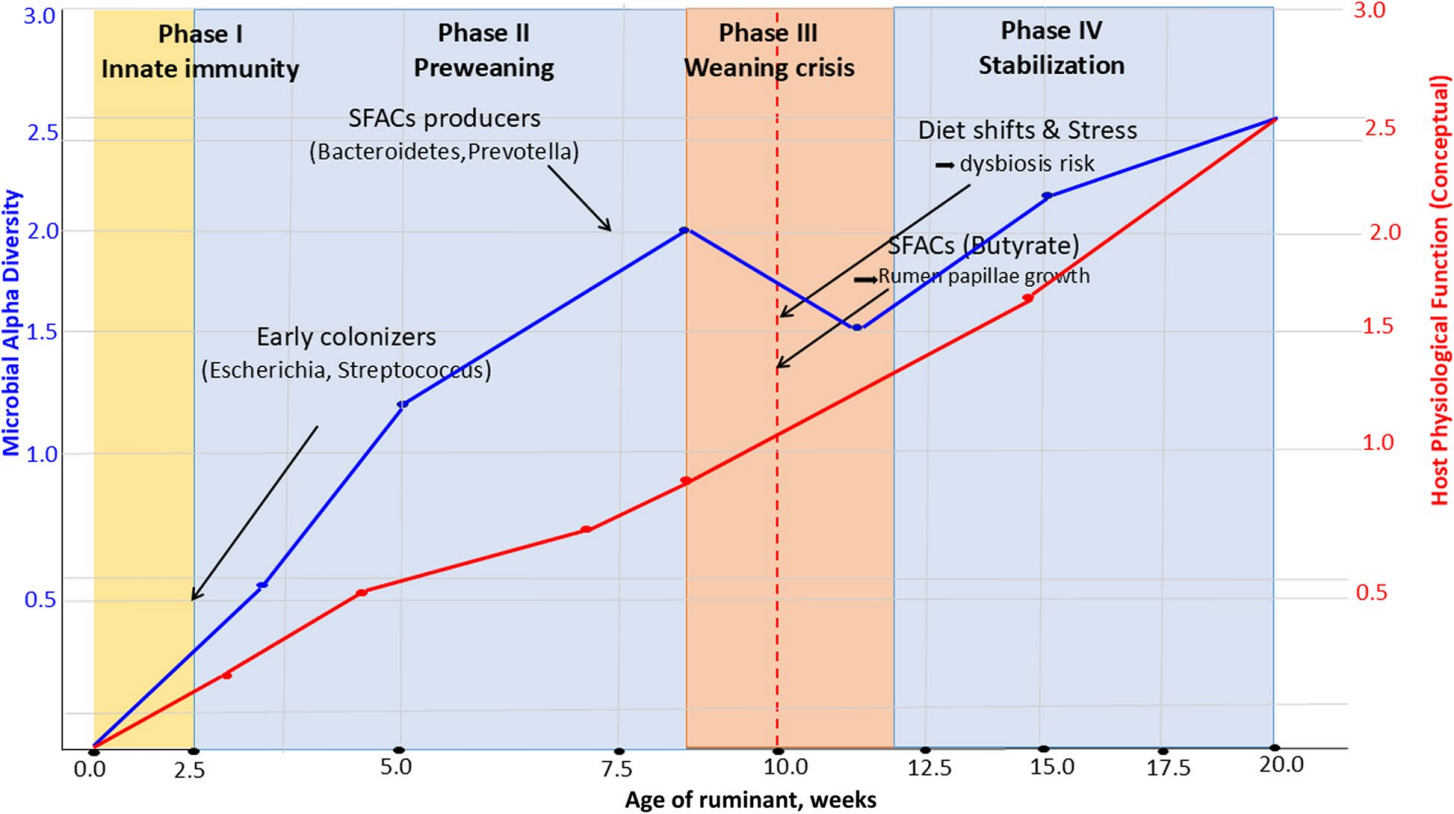
Microbial colonization dynamics and host physiological development in the rumen of young ruminants. This figure illustrates the changes in microbial alpha diversity (blue line) and host physiological function (red line) across different stages of ruminant development (0 to 20 weeks of age)

The gut microbiota maturation in neonatal ruminants is one of the key events that affect gastrointestinal activity as well as the overall health condition [88, 89]. Figure 3 indicates that development occurs in distinct phases with remarkable changes in microbial function and structure from birth to weaning to adulthood. At birth, gut microbiota is largely influenced by maternal sources of microbial exposure, such as the microbiota of vaginal and breast milk, which provide important bacteria for initial colonization [90]. According to Bi et al. [91], the early gut microbes of bottle-fed lambs were dominated by bacteria from the mother’s vagina, ambient air, and the sheep pen floor, 46%, 31%, and 12%, respectively, whereas those of suckled lambs derived from the mother’s teats (43%), and ambient air (28%). Although there is evidence indicating that mammals may be inoculated with a dense microbiota in the uterus, the microbial community undergoes rapid changes in the initial postnatal life [7]. This prenatal colonization hypothesis remains controversial because microbes detected in fetal tissues or amniotic fluid may represent DNA contamination rather than viable colonizers, and their long-term contribution to postnatal gut assembly is still unclear [92, 93]. For instance, *Escherichia* and Clostridia colonize the intestines on the first day after birth, and the diversity in the rectum initially decreases before increasing rapidly after seven days [7]. Nonetheless, some longitudinal studies instead have described a more continuous increase in diversity during the first weeks of life, suggesting that colonization may follow a more deterministic successional pattern [94]. After this period, microbial diversity increases and specific taxa become more prominent around 42 d, which is a prerequisite for the transition to a more complex and stable gut ecosystem [61, 89, 91]. This development continues until the rumination phase (approximately 70 d), whereby the gut microbiota reaches a more stable and diversified form [61]. This process is further stimulated by weaning, which establishes progressive transformations in microbes [95]. At this age, ruminal bacterial populations are more diverse in lambs fed starter diets than in those on milk [96], and the microbiome shifts towards a more adult-like appearance by the age of one year, with families and genera similar to those of adult cattle, but with different operational taxonomic units (OTUs) [14, 97]. However, the long-term significance of these early shifts remains uncertain because some dietary effects on microbiota composition do not persist into adulthood [98].

In addition to the developmental phases, gut microbiota is essential for host metabolism, which performs important processes in amino acid and carbohydrate metabolism that play essential roles in development and health [89]. In contrast, alterations in early life that are triggered by illness or nutritional exposure may lead to substantial structural changes in the intestinal microbiome, which could influence long-term productivity and health [99]. In this regard, microbial interventions, including direct-fed microbes, are a promising approach to normalize gut communities and promote the productivity and health of calves [100]. The disease resistance capability the animals is also improved by a well-established and stable microbiota [101].

### Host genetic regulation of gut microbiota in young ruminants

**Fig. 4:**
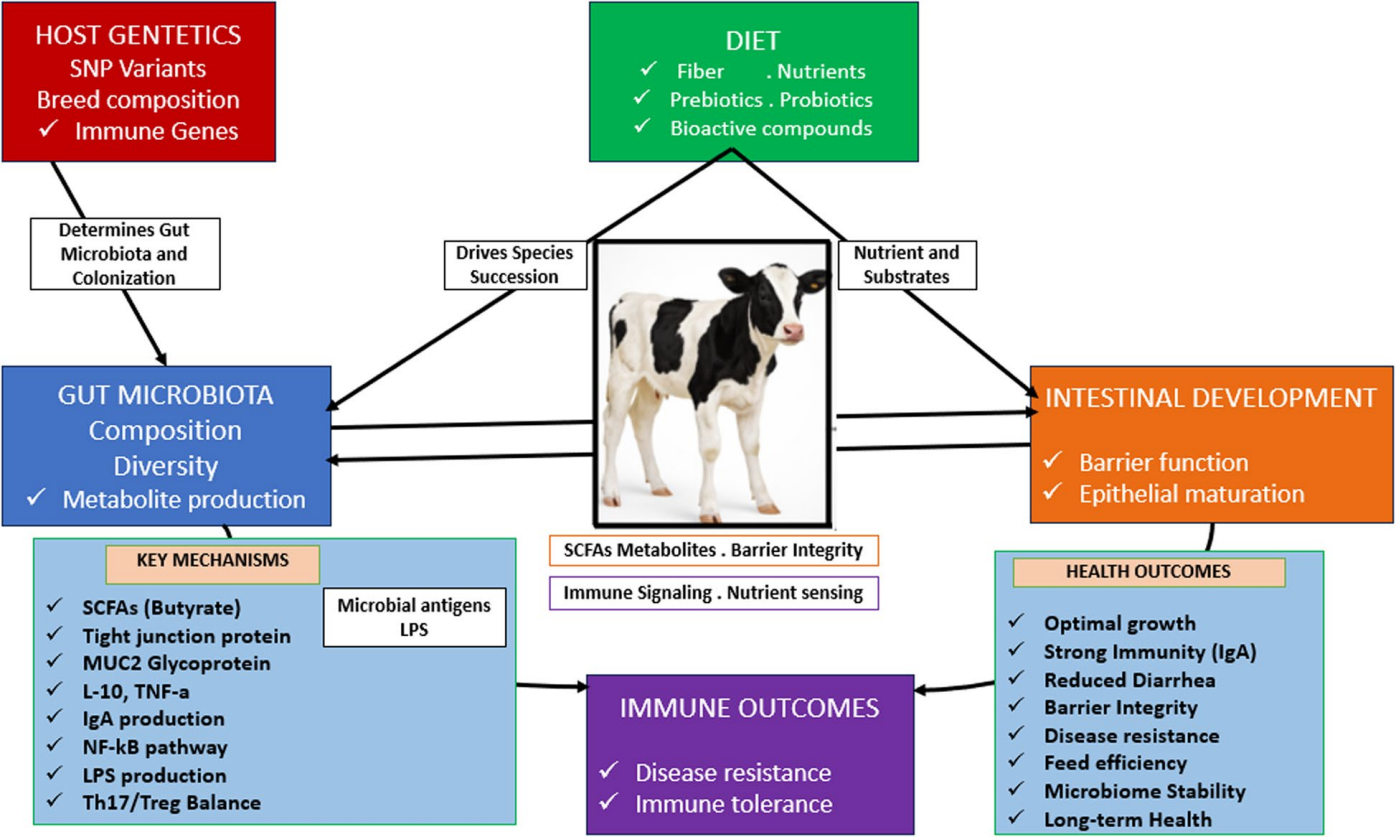
The conceptual integrated framework of host genetics, microbiota, intestinal development, and immune outcomes in young ruminants

Genetic control in the host has a fundamental effect on the structure of the gut microbiota of young ruminants (Fig. 4). It also plays a significant role in shaping the early gut microbiota of calves, with paternal genome and mucin-encoding gene variants associated with bacterial abundance [102]. Moreover, breed composition accounts for more than half of the variation in core bacterial genera in calves, and butyrate-producing bacteria such as *Roseburia* and *Oscillospira* are associated with SNPs in genes involved in immunity and metabolism [103]. Nonetheless, breed-based comparisons have confounded genetic effects with environmental factors, as different breeds often experience distinct management conditions, geographic locations, and nutritional histories. In this context, gut microbiota characteristics are moderately heritable with the heritability estimate (*h*^2^) of 0.38—0.39, and microbial genera and feed efficiency traits share a genetic correlation [104]. However, heritability estimates require cautious interpretation, as they are population-specific and may not translate across different management systems or breeds. Furthermore, the genetic correlation between the microbiota and feed efficiency may reflect pleiotropic effects or linkage disequilibrium rather than direct functional relationships [105]. Genome-wide association has consistently revealed host genomic regions associated with genus abundance, such as *Akkermansia* and *Prevotella*, indicating a genetic basis for microbiota composition [106]. Moreover, host genetics can influence the richness of gut enterotypes that correspond with development and metabolic functions [107]. In beef cattle, for example, some genomic locations have been associated with the richness of the rumen family of bacteria, such as Prevotellaceae and Fibrobacteraceae, indicating that host genes could influence the choice of microbes for maximum energy gain [108]. To this end, Arshad et al. [37] pointed out that transgenesis and the host’s genotype influenced the families of rumen bacteria in neonatal ruminants, with implications for development and gut function. Similarly, host genetics explains approximately 37%–52% of the variation in core bacterial taxa, depending on the growth stage, with environmental and dietary factors contributing significantly to individual differences [103]. Furthermore, the richness and structure of bacterial communities in crossbred and purebred beef calves differ during early life and impact gut microbiota colonization and establishment [109]. However, these GWAS findings have methodological limitations; associated SNPs typically explain only a portion of the phenotypic variance for specific taxa [9], and significant associations with overall diversity metrics are often lacking in large-scale studies.

Moreover, host genetics in sheep, as described by Huang and Li [110], regulate the populations of metagenomic archaea in the rumen and thereby influence methane production and feed efficiency. In dairy calves, the colonization of the core gut microbiota is also regulated by host genotype; there are higher percentages of beneficial bacteria in the hindgut, such as *Faecalibacter* [111]. Similarly, a study on Korean native goats and Hanwoo steers found that the fiber-degrading activity of rumen bacteria is shaped by host-specific and substrate-specific factors, thereby influencing the efficiency of forage utilization in different ruminant species [112]. Likewise, the genetic distinctions in crossbred cattle are also associated with variations in the density of *Prevotella* and *Ruminococcus*, important for fiber digestion [113]. There is also other evidence to suggest that sex and breed may have a potential influence on the relative abundance of Prevotellaceae and other fiber-degrading bacteria, with certain *Prevotella* taxa being positively associated with crossbreeding cattle feed efficiency [114]. Host genetic effects can also be modulated by sex or overridden by diet, particularly for Bacteroidetes members that are primarily diet responsive [9]. These findings demonstrate that the host environment and genetics are powerful predictors of the structure and function of the gut microbiota in young ruminants, with direct impacts on health, growth, and productivity (Fig. 4).

Furthermore, the host genetics contribute in part to regulating microbial colonization and community structure in the gut [115]. The rumen microbiota is also established based on the genetic architecture of the host, thereby determining productivity, state of health, and feed conversion ratio [116, 117]. It has been observed that the distinct microbial community structure observed between monogastric and polygastric species [115], and comparable microbiota has been observed in twin calves raised in a controlled environment [116]. In another study, the correlation between host genetics and rumen microbiota in sheep suggested that the genetic differences affect the colonization and abundance of bacterial genera [118]. Such genetic determinants have great potential in the design of precision interventions to enhance the productivity of livestock [117]. Table 1 indicates that the host genetics affects the gut microbiota of young ruminants. Host genotypes have emerged as one of the most fundamental factors shaping gut microbiota composition and function in ruminants. The rumen microbiota explains 20% of phenotypic variation in Hu lambs [119], whereas in beef cattle, host genetics accounts for 52.2% of the relative abundances of microbiota in the preweaning and postweaning periods [103]. In Holstein and Brown Swiss cattle, host genetics determined 48% of the total microbial composition as measured by both 16S and 18S rRNA analysis [124]. These findings collectively demonstrate that genetic background is a major driver of microbial community assembly. Furthermore, breed type significantly affects bacterial community structure, with microbiota composition greatly varying between different sheep breeds [122], indicating that the genetically determined differences between breeds create distinct microbial ecosystems, even within the same species. The consistency of these genetic effects across multiple cattle breeds and sheep populations suggests a fundamental biological principle: host genetic variation creates selective pressures that favor specific microbial populations over others.

**Table 1:** Influence of host genotype on gut microbial community composition in ruminants

**Experimental unit**	**Age/range**	Methods	**Microbial association**	**Reference**
Hu lambs	1,150 male Hu lambs	GWAS/MWAS	The rumen microbiota explains 20% of the phenotypic variation	[119]
Ji’nan Wild Zoo	Three species belonging to Caprinae	16S rRNA	The relative abundance of intestinal microflora was significantly increased	[120]
Dairy sheep	Multiparous Lacaune dairy ewes	GWAS	Host genetics controls the abundance of rumen microbiota	[121]
Sheep breeds	Three different sheep breeds	16S rRNA	Microbiota composition was greatly different between breeds	[122]
Hainan black goats and Saanen white goats	7–12 months of age	16S rRNA	The intestinal microbiota of black and white goats were slightly different	[123]
Steers and heifers	844 heifers	GWAS	Host genetics affects rumen bacterial community composition	[108]
Beef cattle	228 preweaning calves	16S rRNA	Host genetics and paternal genome significantly affect the gut microbiota of preweaning calves	[102]
Holstein and Brown Swiss	Eighteen animals	16S and 18S rRNA	Host genetics determined 48% of microbes	[124]
Jersey and Holstein cows	5 Holstein and 4 Jersey cows	16S rRNA	Breed type significantly affects the bacterial community	[125]
Beef cattle	278 multibreed Angus Brahman preweaning and postweaning	GWAS	Host genetics accounted for 52.2% the relative abundances of microbiota in preweaning calves	[103]

*GWAS* Genome-wide association study, *16S rRNA* 16S ribosomal RNA, *MWAS* Microbiome-wide association study

### Host age-mediated regulation of gut microbiota

Gastrointestinal development in ruminants is a multifaceted process that is highly dependent on host age (Table 2). Age is a primary factor influencing the composition, diversity, and stability of gut microbiota in young ruminants [90, 136]. These host and age-mediated developmental processes are key to improving the overall performance and productivity of ruminant animals [137, 138]. This age-mediated regulation significantly shapes the gut microbiota, as evidenced by studies showing that microbial diversity in sika deer correlates with age, disease status, and growth performance [128]. Furthermore, transcriptional profiling reveals marked distinctions between neonatal and adult ruminants, particularly in the rumen, with significant changes in gene expression related to pathogen recognition, immunity, and metabolism during the transition from pre-ruminant to functional ruminant [128, 138]. Anatomical development parallels these functional changes; the mass of the rumen, reticulum, omasum, abomasum, and other intestines gradually increases with age [139, 140]. Additionally, the microbial community undergoes dynamic succession. At birth, the rumen and cecum experience a rapid influx of microbes, a community that changes from one month through adulthood [141]. This age-dependent colonization is consistent across species; for instance, the colonization and metabolomic profile within the small intestine of goat kids also vary significantly with age [129]. Moreover, bacterial diversity progresses as calves grow, with Shannon diversity index values notably higher at 21 and 42 d than at 7 d of age, and the variation between individual animals decreases over time [142]. This trend is driven by the combined effects of host maturation and associated dietary shifts, both of which affect microbial diversity from 7 to 49 d of age [143] and 7 to 63 d [144].

**Table 2:** Host age on modulation of microbial colonization

Experimental unit	Age/range	Methods	Results	Reference
Mongolian cattle	At 5, 8, and 36 months of age of fifteen Ujumqin Mongolian cattle	16S rRNA	Age gradually increased the relative abundance of Ruminococcaceae	[126]
Goat kids	Twenty-four newborn kids from born to thirty female goats of similar body weights and age	16S rRNA	The bacterial community compositions in the digesta markedly differed from those in the mucosa across age groups	[127]
Deer	Fifteen neonatal sika deer at 1, 42, and 70 d	16S rRNA	Diversity and richness increased with age	[128]
Goat kids	Twenty-four healthy male goats at birth, 1, 2, and 12 months of age	16S rRNA	A rapid invasion of microbes in the rumen and caecum at birth, and gradually shifts from 1 month of age to adulthood	[129]
Holstein calves and cows	Fifty-eight healthy female Holstein cattle aged 1 week to 5 years old	16S rRNA	Higher microbiota similarities were found in the gut segments of younger animals compared to older animals	[130]
Goat kids	Forty-eight healthy female Laiwu black goats of eight ages (1, 7, 14, 28, 42, 56, 70, 84 days of age)	16S rRNA	In the Jejunum and colon, a significant member of microbiota is observed across all ages	[131]
Holstein’s calves	Fifty-nine calves in two weaning groups at 7 weeks and 17 weeks of age	16S rRNA	A progressive change was observed in the microbiome and metabolome profile after weaning at 7 or 17 weeks of age	[132]
Newborn calves	Twenty-one calves at d 2, d 28, and at weaning	16S rRNA	Significant age-related changes observed in the composition of rumen microbiota	[133]
Newborn calves	From birth to 8 (57 d) weeks old	16S rRNA	The composition of the gut microbiome improved from birth to 8 weeks of age	[134]
Aberdeen Angus calves	Post-birth up to 96 days of age	16S rRNA	↑IL1B, IL33, inflammasome sensor NLRP3, and inflammatory cytokines IL1A	[135]

*NLRP3* NOD-like receptor family pyrin domain-containing three, *16S rRNA* 16S ribosomal RNA, *IL33* Interleukin 33, *L1A* Interleukin 1 alpha, *IL1B* Interleukin 1 beta

Moreover, age is a critical temporal variable influencing the establishment and maturation of ruminant gut microbiota (Table 2). Age gradually increases the relative abundance of Ruminococcaceae in cattle over the first three years of life [126], indicating progressive shifts in the dominant bacterial populations as animals develop. The diversity and richness of sika deer microbiota increase with age from 1 to 70 d [128], demonstrating that microbial ecosystem complexity expands during early development. Bacterial community compositions in digesta markedly differ from those in the mucosa across age groups in goat kids from birth to 30 d [127], suggesting that distinct microbial niches develop as the gastrointestinal tract matures. Notably, higher microbiota similarities were found in the gut segments of younger Holstein cattle than in older animals [130], indicating that microbial community divergence increases as animals grow from young to adult stages. The critical weaning transition at 7 or 17 weeks of age produces progressive changes in both the microbiome and metabolome profiles of Holstein calves [132], highlighting that dietary transitions during development fundamentally reorganize microbial communities and their metabolic capabilities. However, contradictory findings exist regarding the consistency of age-related microbial changes. For instance, there was no significant difference in the relative abundance of major bacterial phyla between different ages in goats [145], suggesting that feeding strategies or weaning age may override simple age effects. Similarly, early weaning in lambs led to decreased rumen microbiota richness and diversity at 26 and 35 d compared to conventionally weaned controls, with partial recovery by 63 d [146], indicating that age effects are highly context-dependent and interact with management practices. Additionally, age is a composite variable encompassing dietary transitions, rumen development, and immune maturation, all of which independently shape microbiota. Thus, age provides a temporal framework rather than a direct causal driver. The convergence of microbial communities with age may result from genetic programming, diet, or microbial competition, but the underlying mechanisms remain unresolved. For example, Recent longitudinal studies demonstrated that fecal microbial alpha diversity (Shannon index) increases progressively from week 1 (3.2 ± 1.1) to week 2 (3.9 ± 1.20) to week 3 (5.2 ± 0.9) in neonatal dairy calves [147], supporting the concept of age-dependent diversification. These challenges emphasize the need for controlled developmental studies, gnotobiotic models, and multi-omics approaches to move beyond associative evidence toward a mechanistic understanding of age-mediated regulation of gut microbiota in young ruminants.

### Regulation of host intestinal development by gut microbiota

**Fig. 5:**
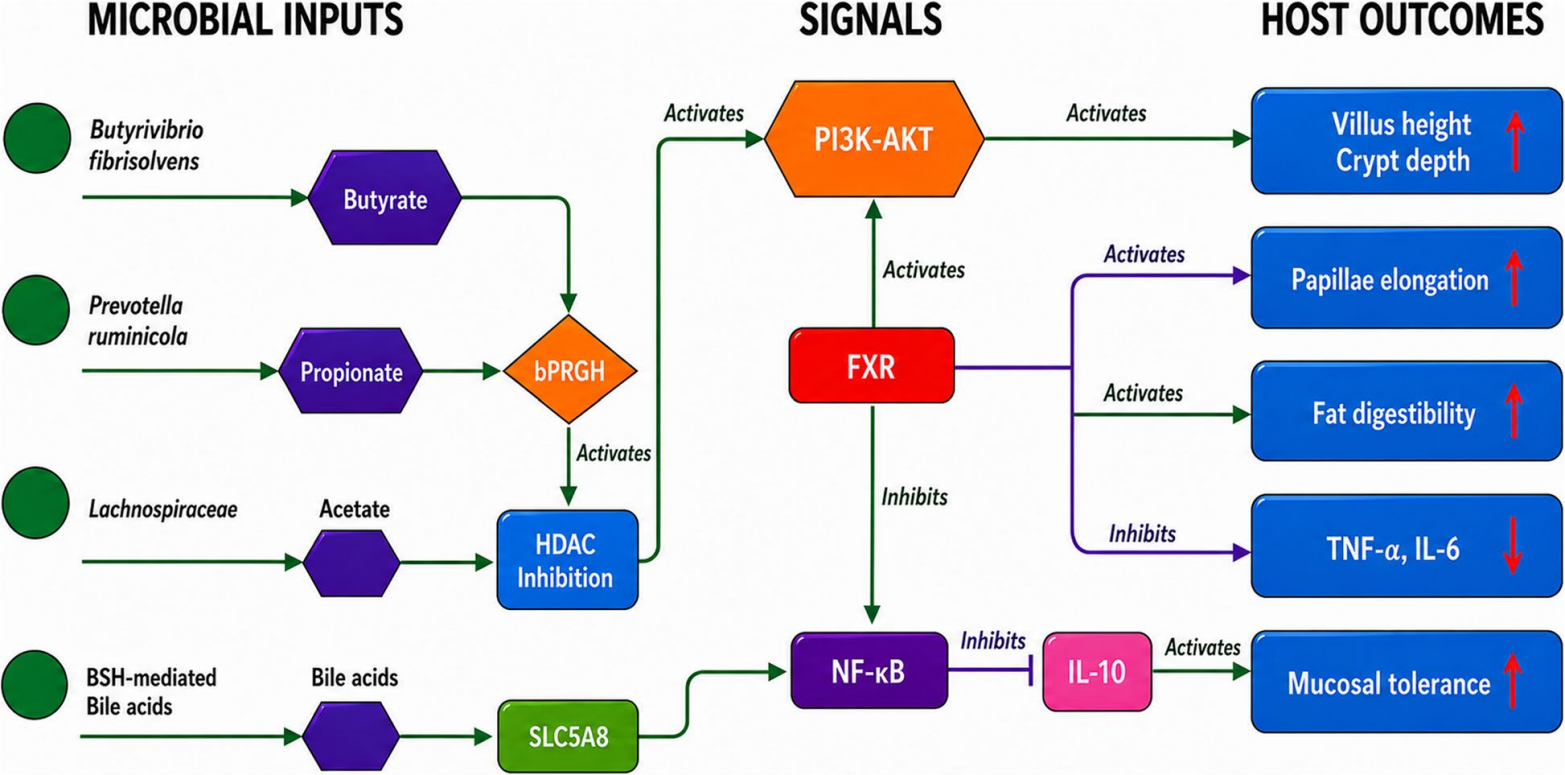
Regulation of host intestinal development by gut microbiota. bPRGH: Bovine prolactin/growth hormone; HDAC: Histone deacetylase; P13-AKT: Phosphoinositide 3-kinase/protein kinase B; FXR: Farnesoid X receptor; NF-kB: Nuclear factor kappa B; IL-10: Interleukin-10; IL-6: Interleukin-6: SLC5A8 (SMCT1): Solute carrier family 5 member 8

The complex interaction of metabolic, immune, and gene-expression mechanisms regulated by the intestinal microbiota affects intestinal development, which is sensitive to dietary and environmental factors. The postnatal period is a critical developmental window characterized by changes in nutrients and absorption and the activation of the host signaling cascade, which enhances the proliferation of epithelia, maturation of immunity, and metabolic adaptation [37, 148]. Early colonizers also generate volatile fatty acids that regulate host gene modules and microRNAs with zinc ion binding and transcriptional control, affecting rumen and intestinal development [6]. These short-chain fatty acids are particularly generated by the fermentation of dietary fibers by microbes [2]. Butyrate mediates epithelial apoptosis and proliferation through G protein-coupled receptor 43 (GPR43) and regulates the expression of the cytokine to maintain mucosal development and stability [149]. Microbial-derived molecules also determine immune homeostasis, building on the formation of gut-associated lymphoid tissue (GALT) and cytokine cues (e.g., IL-1 2, TNF-2, IFN-7), which play vital roles in immune cell differentiation and mucosal defense [150]. Diet is another important factor in providing beneficial bacterial taxa, including Lachnospiraceae and Ruminococcaceae, which create an immune threshold, and high-starch-based diets break the immune balance and create an inflammatory environment based on TH2-secreted cytokines [151]. In addition, metabolism and immunity, multi-omics research studies have shown that microbial colonization restores host transcriptomic and epigenetic topography [152]. These maintenance processes are region-specific, along with different gastrointestinal segments. Replication and backup mechanisms become activated in the small intestine and involve a peak activity in carbohydrate and amino acid metabolism in the large intestine [61]. Microbial succession is strictly connected with the developmental stage; the functional replacement and hub portions of microbes support metabolic adjustment and immune development [148]. Figure 5 indicates the regulation of host intestinal development by gut microbiota.

### Regulation of host mucosal immunity by gut microbiota

**Fig. 6:**
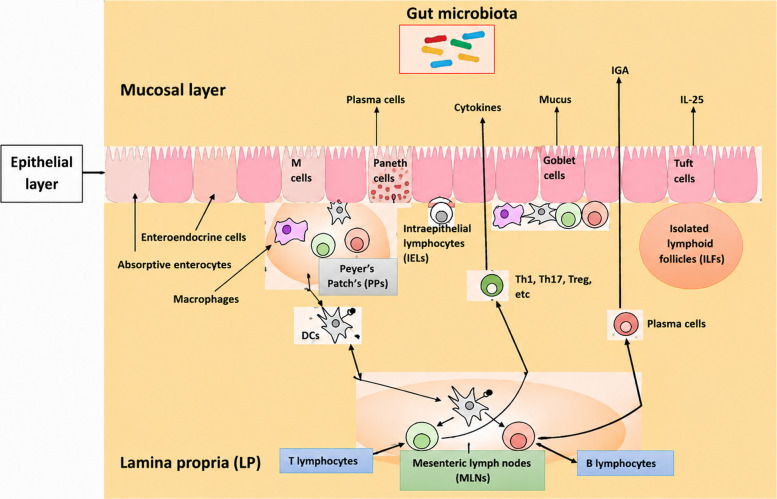
The composition of the intestinal mucosal immune system. The mucus layer, the epithelial layer, and the intestinal mucosal immune system make up the intestinal mucosal immune system. It forms the mucus layer as the main physiological barrier, a combination of molecular signals of the cells of intestinal epithelium, which are mucins, antimicrobial peptides (AMPs), and IL-25, and T lymphocyte-produced cytokines and IgA produced by B lymphocytes, assists in protection against pathogenic infection and invasion [157]

Gut microbiota has become a key controller of mucosal intestinal immunity in younger ruminants, and immunological progression is restricted to a narrow zone, as determined by previously set microbiota signals; it depends on the diet and relationships with the environment [32, 153]. Primary colonization take place through the colostrum, the milk, and the exposure to the environment, providing the host with the necessary microbial consortium necessary to conduct immunological programming [90, 91, 154, 155]. A diverse microbiota then turns out to be an immunomodulatory metabolic organ, and it generates the necessary insulinogenic metabolites, such as SCFAs and bile acids [156, 157]. These metabolites regulate innate and adaptive immune reactions and enhance defenses against enteric pathogens and reinforce the intestinal barrier, creating the preconditions of host health and disease resistance [2, 37, 158]. Microbial maturation is a fragile process prone to dysbiosis, which can be easily disturbed by perturbations. Such dysregulation contributes to disease development, which is further exacerbated by additional adversity, e.g., infectious agents, poor nutrition, or management-related stressors [101, 159]. The pathophysiology of neonatal diarrhea and cryptosporidiosis is characterized by changes in the composition of the microbial network, including increased Enterobacteriaceae, decreased metabolite production, and shifts in immunological markers, such as increased immunoglobulin levels and elevated cytokine levels [160]. These mechanisms are also governed by direct associations between individual bacterial taxa and individual host immune gene expression, highlighting the extreme impact of microbial communities on the immunological condition of the host [161, 162]. Consequently, early-life interventions and the implementation of strategies to regulate microbial colonization are a top priority in promoting health [131]. The use of administered probiotics, probiotic cultures, and fortified colostrum, as well as specific nutritional interventions, has been successful in increasing microbial diversity, enhancing beneficial metabolic output, and eventually improving immunity and the treatment of diarrhea [163–165]. In summary, achieving balanced and stable gut microbiota by providing specific interventions is a vital approach that must be implemented to optimally develop the immune system in young ruminants [165–167]. The mucus layer, the epithelial layer, and the intestinal mucosal immune system make up the intestinal mucosal immune system, as indicated in Fig. 6.

## Dietary regulation of gut microbiota and host intestinal development

### Milk replacer and starter

Early nutrition, delivered through milk replacer and starter feed, simultaneously shapes gut microbiota, immune competence, and later performance-controlling metabolic set points in young ruminants. Milk replacers tailored to replicate maternal milk stimulate Lactobacillus and Bifidobacterium and repress enteric pathogens [168]. Oligosaccharide-enriched milk replacer promotes *Bifidobacterium* and *Faecalibacterium prausnitzii* colonization [169]. However, the relative contributions of oligosaccharides versus protein sources remain undetermined, and confounding factors under different feeding regimens complicate causal interpretation; associations do not establish that specific milk replacer components directly cause bacterial enrichment. Starter-feed introduction also accelerates colonization of fibrolytic taxa (*Prevotella, Ruminococcus)*, boosts ruminal concentrations of SCFAs, and stimulates growth of the epithelial papillae [170]. The physical structure, nutrient density, and additives, such as yeast or phytogenic products, all promote a microbial balance toward beneficial microbes and away from pathogens [168], whereas overfeeding milk replacer with no step-down regimen depresses rumen maturation. Balancing the two rearing stages, sequential substitution of milk replacer with textured starter feed facilitates sequential microbial succession, maximizes rumen performance, and boosts feed efficiency [169, 171]. Because SCFAs and other microbial catabolites also serve as signaling molecules for host immunity and metabolism [172], research has increasingly focused on non-antibiotic additives that enhance rather than disrupt these pathways without selecting for antimicrobial resistance [173]. In general, an accelerated yet stepwise regimen of milk-to-starter offers a practical, non-drug option for achieving rapid growth of the rumen and ensuring long-term growth performance in pre-weaned calves.

Dietary supplementation with milk replacer and starter feed significantly modulates microbial community structure and function in young ruminants, with lasting consequences for animal health and performance (Table 3). Increasing the milk replacer oligosaccharide content increases *Bifidobacterium* and *Faecalibacterium prausnitzii* in Holstein–Friesian dairy calves [169], demonstrating that specific dietary components directly promote beneficial bacterial colonization. Butyrate-fortified milk replacer increases populations of *Butyrivibrio* and short-chain fatty acid production in the colon of Holstein–Friesian bull calves [175], establishing that metabolic byproducts enhance fermentation capacity and energy harvest efficiency. In yaks, milk replacer supplementation increases *Prevotella* and fiber-degrading enzymes while decreasing Actinobacteria, improving rumen function, and reducing disease risk [176]. As a starter diet with alfalfa increases *Coprococcus*, *Pseudobutyrivibrio*, and other fermentation-associated bacteria in yak calves, reducing intestinal inflammation and enhancing immune function [150]. These findings establish that early nutritional interventions during the critical milk replacer transition period establish the foundational microbial communities that support subsequent ruminant development and productivity.

**Table 3:** Milk replacer and starter modulate the gut microbiota of young ruminants

Species/animals	Intervention/factor	Microbial changes/findings	Functional outcomes	Reference
Yimeng black goats	Milk replacer	Alters gut microbiota composition; ↑ Lachnospiraceae, Ruminococcaceae, *Eubacterium*, ↓ *Porphyromonas*, Enterococcus, *Clostridium*	Maintains stability, reduces pathogens, supports function	[174]
Holstein–Friesian dairy calves	MR oligosaccharide content	↑ *Bifidobacterium*, *Faecalibacterium prausnitzii* with conjugated OS; OS content shapes early microbiota	Enhanced probiotic colonization, gut health	[169]
Holstein–Friesian bull calves	Butyrate-fortified MR	↑ *Butyrivibrio, Shuttleworthia*, *Phascolarctobacterium*; ↑ SCFA in colon	Improved fermentation, gut health, and growth	[175]
Yak calves	MR in yaks	↑ *Prevotella*, ↑ fiber-degrading enzymes, ↑ Bacteroidetes, ↓ *Actinobacteria*	↑ VFA, improved rumen function, reduced disease risk	[176]
Hu lambs	MR feeding level	High MR: ↑ diversity, ↓ fiber-degrading bacteria, ↓ starter intake	Short-term growth ↑, long-term rumen development ↓	[177]
Yak calves	MR + probiotics	↑ Bacteroidota, Proteobacteria, and *Ruminococcus*	Rumen development, ↑ VFA, ↑ body weight	[168]
Yak calves	Starter + alfalfa	↑ *Coprococcus*, *Pseudobutyrivibrio*, *Flavonifractor*, *Synergistes*, *Sutterella*	↓ Inflammation, ↑ immune function, ↑ fermentation	[150]
Dairy calves	MR feeding frequency	Once vs. twice daily: no change in diversity/richness, similar metabolome	Flexible management, stable microbiota	[178]
Holstein dairy calves	MR + ethoxyquin	MR + ethoxyquin: ↑ *Bacteroides*, *Alloprevotella*, ↓ *Alistipes*, Ruminococcaceae	↑ Starter intake, antioxidative ability, and growth	[179]
Dairy × beef crossbred calves	MR + *Macleaya cordata*	Minor changes in microbiota, ↑ rumen papillae length, ↑ villus height, and crypt depth	Enhanced gut morphology and development	[67]
Yimeng black goats	MR supplementation	↑ Verrucomicrobia, *Akkermansia*, *Veillonella*, *Ruminococcus*; ↓ *Actinobacteria*, *Proteobacteria*, *Turicibacter*	Improved growth, rumen microbiota balance	[180]
Holstein calves	Sodium butyrate in MR	↑ Ruminal bacteria load, ↑ VFA, ↑ digestibility, improved fermentation	Enhanced microbial development, feed efficiency	[181]
Holstein bull calves	Botanical/DFM starter	↑ *Lactobacillus* improved gut health, altered DMI, and growth	↑ Growth, ↓ scours, variable performance	[182]

*MR* Milk replacer, *ARGS* Antimicrobial resistance genes, *DFM* Direct-fed microbe, *VFA* Volatile fatty acids, *DMI* Dry matter intake

### Forage concentrates ratio

The dietary forage-to-concentrate ratio is a critical factor modulating gastrointestinal microbiota in young ruminants (Table 4). Substantial evidence demonstrates that high-fiber forages promote a fibrolytic microbial community. For instance, feeding a high-fiber diet to cows increased *Rumenomonas* and decreased *Megasphaera elsdenii* [200], whereas alfalfa hay increased the abundance of fibrolytic bacteria in neonatal lambs [165]. Such forage-based diets also improved beneficial metabolite enrichment and associated metabolic pathways [194]. Conversely, concentrate-rich diets markedly shifted the microbial ecosystem towards taxa that utilize fermentable carbohydrates. Starter feed in pre-weaning calves increases the abundance of *Megasphaera*, *Sharpea*, and *Succinivibrio* [201], and solid diets amplify rumen and epithelial microbiota in goats [202]. Increasing dietary grain from 0 to 50% in goats also changes ruminal microbiota composition [203]. In another study, concentrate supplementation significantly decreased microbial diversity throughout the gut composition in young calves [204]. In addition, specific supplements, such as a concentrate mixture with butanoic acid isopropyl ester, decreased ruminal butyric acid while increasing ammonia-producing and sulfate-reducing bacteria [36], and dietary protein or urea supplementation increases the diversity and abundance of beneficial rumen bacteria in lactating ewes and Hu lambs, respectively [205, 206]. However, protein supplementation effects are inconsistent: lambs fed 111.7–143.6 g/kg DM crude protein showed no effect on *Ruminococcus albus*, *R. flavefaciens*, or *Fibrobacter succinogenes* [207], possibly reflecting differences in protein degradability or carbohydrate synchronization. The early-life diet is a primary driver, as the total microbial community and its initial establishment in dairy calves were significantly influenced by dietary differences [203], with even early feed restriction affecting ileal epimural microbiota development during suckling [208]. Microbial variation has also been observed between suckled and bottle-fed lambs [91]. However, not all studies report significant changes; one study found no difference in microbial composition in cows subjected to varying forage-concentrate ratios [199], suggesting that host breed and physiological state may account for these discrepancies. These studies revealed that dietary modifications consistently alter the composition, structure, and fermentation patterns of the host microbiota [209].

**Table 4:** Forage concentrate ratio modulates the gut microbiota of young ruminants

Experimental unit	Methods and treatments	Results	Reference
Da’er goats	30 animals fed basal diet, basal + 0.12% coated methionine (CM), or basal + 0.22% butanoic acid isopropyl ester (HMBi)	HMBi ↑ BW, FI, and feed-to-gain ratio, and both CM and ↓ rumen butyric acid concentration	[36]
Sheep	Sheep fed corn straw, alfalfa, and caragana diets	The rumen, ileum, and cecum microbial composition and structure were significantly changed in the sheep fed the Cargana diet	[183]
Hu sheep	Male sheep (*n* = 15) were assigned to CON, non-pelleted high-grain, and pelleted high-grain diets	High-grain diet ↑ composition of commensal bacteria in the colon and the richness of *Olsenella, Alloprevotella*, and *Ruminococcus*	[184]
Dairy cows	Treatments: Control (HFLO), Induction (LFHO), and Recovery (HFLO)	The proportion of several bacterial genera was significantly enriched following the induction of milk fat depression (MFD)	[185]
Rambouillet, Hampshire, and Suffolk sheep breeds	Seventy-seven animals under two different diets: a forage-based diet and a concentrate-based diet	Sheep fed a forage-based diet had greater microbial diversity than concentrate-fed animals	[186]
Holstein dairy cows	Forty-five cows’ GIT microbiota analyzed using Illumina amplicon sequencing	Early-life diet affects the initial microbial community establishment	[97]
Goat	Nine 2-month-old calves were assigned to three groups with 85%, 100%, and 130% of standard diet DE and CP levels	↑ Nutritional level, intestinal microbiota's growth performance, and composition ↓ richness of the SCFAs-producing bacteria in the colon	[187]
Newborn lambs	Two groups: control diet and additive diet (milk replacer + BETAFOS60 + garlic residues)	Additive improved the richness of *Bifidobacteria, Enterococcus, Lactobacillus, *and* Veillonella*	[188]
Dairy calves	32 animals were fed CON, CON + *Saccharomyces cerevisiae boulardii* (SCB), CON + *Lactobacillus acidophilus* (LA), or CON + antibiotics (ATB) for 96 d	Both SCB and LA ↓ pathogenic bacteria genera and ↑ beneficial bacteria, and ATB had a higher influence on the ileum bacterial composition	[189]
Lambs	90 animals allocated to T1 (native grass hay, HA), T2 (pelleted native grass hay, GP), or T3 (pelleted native grass hay + concentrate, GPC)	GP and GPC diets ↑ growth performance, and the rumen microbiota varied in response to feed types	[190]
Steers	Fifty animals were assigned to five treatments with Starea (starch-urea): T1 SU0 (0%), T2 SU8 (8%), T3 SU16 (16%), T4 SU24 (24%), T5 SU32 (32% of urea-N in total N)	Starea influences the composition and structure of intestinal bacteria	[191]
Guanling yellow cattle	Twelve animals were assigned to a basal diet (BD) and a Jiang-flavor (DDGS) diet, replacing 25% of the concentrate	DDGS diet ↑ microbial structure and metabolome in ruminal and cecal contents	[192]
Calves	9 calves assigned to GF (concentrate), GFF (alfalfa:oat grass 3:2), and TMR (concentrate:alfalfa grass:oat grass)	A TMR ↑ rumen digestive enzyme activities, nutrient absorption, and microenvironment balance	[157]
Hu sheep	Fifteen animals were assigned to CON, HG (non-pelleted high-grain), and HP (pelleted high-grain)	High-grain diets ↑ the abundance of acid-producing microbiota in the ileal mucosa and ↓ probiotic species	[193]
Holstein calves	Thirty animals were allocated to short oat hay (SO) and short soybean hull (SS)	SO ↑ enrichment of beneficial metabolites and metabolic pathways, expression of genes, and development of rumen epithelium	[194]
Aberdeen Angus and Limousin	Beef cattle were fed different ratios of concentrate and forage diets	Concentrate-based diet ↑ relative abundance of Proteobacteria	[195]
Dairy cows	Cows feed on different ratios of fiber (88%, 76%, and 57.5%)	Eighty-eight percent of a fiber diet ↑ fibrolytic and cellulolytic bacteria	[196]
Nellore feedlot steers	Four different roughage and concentrate ratios	*Selenomonas ruminantium* and *Megasphaera elsdenii, Lactobacillus* sp.*,* and *Streptococcus bovis* increased, while *Fibrobacter succinogenes, Ruminococus flavefaciens* and *Ruminococcus albus* decreased in the rumen by a high-concentrate diet	[197]
Yaks and goats	Two ruminants, goats and yaks, in captive and free-range modes	The free-range ruminants’ gut microbial diversity was higher than that of captive ruminants	[198]
Holstein–Friesian cows	Twelve animals assigned to control forage: concentrate ratio (45.4:54.6) and (24.8:75.2) diets	The microbial composition did not differ between treatments	[199]

*GIT* Gastro-intestinal tract, *TMR* Total mixed ration, *SO* Short oat hay, *SCFAs* Short-chain fatty acids, *DDGS* Distillers dried grains with soluble, *CM* Coated methionine

Furthermore, the dietary forage-to-concentrate ratio exerts a powerful influence on microbial community composition and, by extension, on the metabolic capacity of the rumen ecosystem (Table 4). High-grain diets increased the composition of commensal bacteria in the colon and enhanced the richness of *Olsenella, Alloprevotella*, and *Ruminococcus* in Hu sheep [184], indicating that rapidly fermentable carbohydrates select for specific bacterial populations adapted to high-energy substrates. In contrast, sheep fed forage-based diets had greater overall microbial diversity than concentrate-fed animals in multiple breeds [186], suggesting that structural plant material supports a broader range of specialized fiber-degrading microbes. Concentrate-based diets increased the relative abundance of Proteobacteria in beef cattle [195], whereas high-fiber diets enriched fibrolytic and cellulolytic bacteria in dairy cows [196]. These compositional shifts reflect fundamental changes in microbial metabolic pathways: high-concentrate diets favor rapid fermentation and organic acid production, while high-forage diets support structural carbohydrate degradation and volatile fatty acid production. A total mixed ration increased rumen digestive enzyme activities, nutrient absorption, and microenvironment balance in calves [157], indicating that optimal forage-concentrate ratios maximize both microbial and host enzymatic capacity for nutrient extraction. In general, the inability to control confounding factors, feed particle size affects rumen pH and cellulolytic bacteria independent of forage type and individual genetic variation, means observed associations may reflect physical diet characteristics rather than nutritional composition.

### Probiotics as modulators of the gut microbiota

Probiotics are communities of microorganisms that are essential for improving gut microbiome health, preventing digestive disorders, and enhancing overall well-being. Common probiotics are primarily derived from lactic acid bacteria such as *Bifidobacterium* and *Lactobacillus* [210]*.* In growing Holstein–Friesian calves, the supplementation of lactobacillus-based direct-fed microbes improved weight, gut development, and microbial diversity [100]. Nevertheless, contradictory findings across studies highlight substantial variability in probiotic efficacy. While some studies report that *Lactobacillus acidophilus* supplementation improves growth and reduces scouring, others have found that *Bacillus*-based probiotics in milk replacer did not influence health or growth, starter intake, and fecal bacterial counts [211]. These inconsistencies demonstrate that probiotic effects are highly strain-specific, dose-dependent, and context-sensitive, with no universal benefit across all scenarios [212]. Furthermore, the supplementation of concentrated kids’ diets with varying levels of probiotics showed a substantial increment in bacterial counts (76%) from 10.50 × 10^9^ CFU/mL before feeding to 18.50 × 10^9^ CFU/mL at the third hour after feeding [213]. Correspondingly, supplementation of 20 mL *Lactiplantibacillus plantarum *TSD10 per day to Ongole cattle compared to the control, in the abundance of *Ruminococcus albus* rose (from 9.88 to 12.62 CFU/mL) [214]. In another study, the calves offered 2 × 10^10^ CFU/g, *L. acidophilus *(1 g/calf/d), improved the population of favorable gut microbiota [215]. A study in lambs indicated that the increase in *Lactobacillus* and *Bifidobacterium* observed following the supplementation of Enzimsporin™ probiotics [216]. However, in similar studies, potentially harmful bacteria such as *Enterococcus, Escherichia coli,* and *Yeast* were decreased, which maintained the good gut health of lambs. In a study, *Lactobacillus* administration also significantly affected the total bacterial community composition of pre-weaned dairy calves, such as *Bifidobacterium* and *Akkermansia* [217]*.* Similar effects were also observed in Boer and speckled goat breeds, where the administration of *Lactobacillus rhamnosus* and *Enterococcus faecalis* favoured the survival of beneficial microbial populations in the rumen [218]. Moreover, the dietary inclusion of probiotics increased the relative abundances of *Ruminococcus, Succiniclasticum,* and *Acidaminococcus* in lambs [219]. Ewes fed *Bacillus amyloliquefacien*s H57 also had a significant effect on the rumen microbial community structure compared to the control diet [220].

The effects of probiotics on the gut microbiota of young ruminants are summarized in Table 5. In young ruminants, probiotic interventions consistently improve intestinal barrier integrity, boost immunological responses, and increase beneficial microbial populations, whether through direct-fed microorganisms or bacterial strains. In newborn Holstein calves, lactobacillus-based probiotics improved the ratio of Bacteroidota to Firmicutes [221], creating a more favorable microbial community structure linked to better metabolic health. In the jejunum and ileum of Chinese Holstein calves, probiotic supplementation with *L. reuteri* and *L. johnsonii* enhanced villus height to crypt depth ratios and tight junction gene expression while lowering pro-inflammatory cytokines [223], indicating that probiotics fortify the intestinal mucosal barrier and foster immune tolerance. Probiotics and medical plant extracts enhanced plasma antioxidant activity and decreased weaning age in Holstein calves [225], demonstrating the synergistic benefits of beneficial bacteria and botanical chemicals. Red blood cell counts, total protein, albumin, and hematocrit concentrations were elevated in male Holstein calves when *Bacillus toyonensis* and *Lactobacillus plantarum* were combined [227]. This suggests that probiotic-induced improvements in microbiota function improve systemic nutrient bioavailability and blood protein status. In Holstein bull calves, *Saccharomyces cerevisiae boulardii* selectively increased the populations of *Bifidobacterium*, *Lactobacillus*, and *Faecalibacterium prausnitzii* [229], demonstrating that specialized probiotic strains give beneficial taxa an advantage over harmful bacteria. Together, these results demonstrate that probiotic supplementation during crucial developmental windows produces a cascade of advantages, including increased intestinal barrier integrity, decreased inflammation, improved microbial community structure, and improved systemic health indices.

**Table 5:** Probiotics on the modulation of gut microbiota of young ruminants

Experimental units	Numbers	Methods and treatments	Results	Reference
Holstein newborn calves	42	Two *Lactobacillus*-based probiotics (6BZ or 6BY) independently and in combinations	↑Bacteroidota:Firmicutes and Proteobacteria ratios, growth performance, and gut microbiota profile	[221]
Newborn Holstein calves	36	Control, *E. coli* O78:K99 C gentamycin; and *E. coli* O78:K99 C supplemental MSP	MSP ↑ the fungal richness and diversity (Chao1 and Shannon indices), along with jejunal mucosal expression of ZO-1, and occludin mRNA	[222]
Chinese Holstein calves	40	Control; *L. reuteri* L81 (2 g/d/calf); *L. johnsonii* L29 (2 g/d/calf); G4 composite *L. reuteri* L81 + *L. johnsonii* L29 (2 g/d/calf)	The probiotics ↑ ratio of villus height to crypt depth in the ileum, gene expression of the tight junction, and the richness of beneficial bacteria ↓ *INF-γ* and *IL-8* gene expressions in the ileum and jejunum mucosa, respectively	[223]
Newborn Holstein calves	14	Control group: normal saline; treatment group (TG): 40 mL of the compound	TG ↑ IgA, IgG, and IgM immune indexes by 10.44%, 4.85%, and 6.12%, respectively, and the abundance of beneficial strains	[224]
Newborn Holstein calves	30	Control (C) calf starter + whole milk; C + 2 g probiotic; C + 1.5% medical plant; C + 1.5% medical plant +2 g probiotic; control +3% medical plant; C + 3% medical plant +2 g probiotic	1.5% medical plant ↑DMI, plasma antioxidant activity, performance, the immune system, and ↓ the weaning age of calves	[225]
Holstein calves	12	G1: 1.3% NaHCO_3_ + 0.9% NaCl + routine diarrhea treatment; G2: same + oral probiotic supplement	↑Total antioxidant (TAS) in the treatment group	[226]
Holstein calves	24	Group 1: untreated control group, and Group 2: 1 × 10^10^ CFU per half (the LP299v group)	LP299 ↑ plasma concentration of immunoglobulin G, concentrations of glucose, total superoxide dismutase, immunoglobulin A, interferon-γ, and soluble CD4, and growth performance, antioxidant, and immune capacity	[123]
Newborn Holstein male calves	32	Four groups: CON; BT (*B. toyonensis*, 3 × 10^9^ CFU/calf/d); LP (*L. plantarum*, 1 × 10^10^ CFU/calf/d); LP + BT (half dosages of each)	LP + BT ↑ RBC counts. The LP + BT group ↑ TP, albumin, globulin, and hematocrit concentrations	[227]
Holstein calves	50	CG: milk replacer; TG: milk replacer +7.5 mL *B. subtilis* probiotic per calf/d	*B. subtilis* ↑ growth performance, BW at 30, 60, and 90 days of age, and phosphorus concentration at 30 d, 9.36% (2.99 mmol/L to 3.27 mmol/L )	[228]
Holstein bull calves	20	Control group; TG: *Saccharomyces cerevisiae boulardii* (SCB)	SCB ↑ *Bifidobacterium*, *Lactobacillus*, and *Faecalibacterium prausnitzii*	[229]
Holstein calves	12	T1: Control; T2: *Saccharomyces cerevisiae*, *Aspergillus oryzae*, *Kluyveromyces marxianus* + 0.5 g/kg concentrate; T3: Same microbes + 1 g/kg concentrate	T1 ↓ ADG and ↑ fecal compared to the supplemented group. Probiotics ↑ β-hydroxybutyrate and total volatile fatty acids of ruminal fluid	[230]
Gyr × Holstein dairy calves	90	Three groups: CON (*n* = 30), whole milk; BAC (*n* = 30), milk +1.0 g *Bacillus*-based DFM/d; MIX (*n* = 30), milk +1.0 g *Bacillus* DFM +1.2 g *Enterococcus faecium* 669/d	DFM ↑ weaning BW, growth rates, while also modulating the prevalence of bacteria and protozoa	[231]
Calves	12	T1: supplemented with (treated) and T2: without probiotics feed (Control)	T2 ↑ plasma IgG (ng/mL) and cholesterol levels, fecal characteristics, ↓*E. coli* in feces, diarrheal incidence, and ↑ immunoglobulin	[232]
Calves		Two groups of six animals were formed according to the principle of analogies	T “Immunobacterin-D” accelerates the population of microbiota populations compared to calves in the control group	[233]
Newborn Holstein calves	48	Four groups: (1) CON; (2) EFE-treated wheat straw + probiotic; (3) EFE-treated wheat straw only; (4) EFE-treated wheat straw + probiotic	EFE ↑ FE, ADG (150 g/d), FW (11.3 kg), NDF digestibility, and ↓ the ratio of acetate to propionate in the rumen	[234]

*IgA* Immunoglobulin A, *IgG* Immunoglobulin G, *IgM* Immunoglobulin M, *ADG* Average daily gain, *NDF* Neutral detergent fiber, *CFU* Colony forming unit, *EFE* Exogenous fibrolytic enzyme, *DFM* Direct-fed microbials, *BW* Body weight, *MIX* Mixture, *RBC* Red blood cells, *CG* Control group, *MSP* Microbially-enhanced soy protein

However, previous studies on probiotics have rarely evaluated their persistence beyond the end of supplementation. Another major challenge has been the practical use of the product, particularly its viability during storage, its stability when mixed into the feed, and its ability to survive passage through the abomasum to reach the target site. Additionally, microbial inoculations do not quantify the administered strains within the animal and how their abundance changes over time. Long-term investigations are also lacking, as such effects cannot be captured in short-term studies. Moreover, past studies have often relied on the enumeration of supplemented microorganisms by culture or sequencing of the 16S rRNA gene; however, such approaches cannot differentiate between supplemented and native strains. In many studies, confounding variables have not been controlled, meaning that the observed associations might reflect the effects of control measures rather than the true impact of the probiotics. Lastly, considering these factors for future studies will improve the effect of different probiotic strains on the performance of the animal.

## Summary and future perspectives

Regulation of the host microbiota in young ruminants is a dynamic and multilayered process governed by genetics, nutrition, immunity, and management. Inherited host factors place the first limits on microbial colonization, and epigenetic and metabolic feedback loops adjust community assembly to dietary changes. These pathways can be altered with early-life interventions, such as maternal supplementation, to provide lifelong benefits in terms of efficiency and health. This early phase of the microbial community develops in relation to diet and the environment and plays a critical role in regulating the immune system of young ruminants. Future research should prioritize several specific areas. First, identification and validation of microbial metabolite biomarkers, especially short-chain fatty acids tied to intestinal development and immune maturation in young ruminants, is essential. Second, determining the optimal timing and dosage of early-life interventions is key to sustaining benefits for physiology and production. Third, integrating host genomics with microbiome manipulation strategies enhances the precision and effectiveness of microbiota regulation approaches. Additionally, the development and application of strain-specific tracking methods to accurately quantify the abundance and persistence of administered probiotic strains within the host gut environment is crucial, as current CFU-based enumeration underestimates the viability and species-level detection.

## Data Availability

The paper dataset prepared for this review is available in an Excel file and will be made available upon reasonable request.

## Abbreviations

ADG: Average daily gain
BW: Body weight
CFU: Colony forming unit
CM: Coated methionine
CP: Crude protein
DDGS: Distillers’ dried grains with soluble
DE: Digestible energy
DFM: Direct fed microbials
EFE: Exogenous fiberolytic enzymes
GALT: Gut-associated lymphoid tissue
GIT: Gastrointestinal tract
GWAS: Genome-wide association study
IgA: Immunoglobulin A
IgG: Immunoglobulin G
IgM: Immunoglobulin M
IL1B: Interleukin 1 beta
IL33: Interleukin 33
IL1A: Interleukin 1 alpha
MSP: Microbially enhanced soy protein
MWAS: Microbiome Wide Association Study
MIX: Mixture
NDF: Neutral detergent fiber
NLRP3: NOD-like receptor family pyrin domain-containing
OTUs: Operational taxonomic units
PRISMA: Preferred reporting items for systematic review and meta-analyses
PubMED: Public medline
RBC: Red blood cells
rRNA: Ribosomal ribonucleic acid
SCFAs: Short-chain fatty acids
VOS: Visualization of similarities

## References

[1] Kaczmarczyk M, Löber U, Adamek K, Węgrzyn D, Skonieczna-Żydecka K, Malinowski D, et al. The gut microbiota is associated with the small intestinal paracellular permeability and the development of the immune system in healthy children during the first two years of life. J Transl Med. 2021;19(1):177. 10.1186/s12967-021-02839-w. 33910577 10.1186/s12967-021-02839-w PMC8082808

[2] He Z, Dong H. The roles of short-chain fatty acids derived from colonic bacteria fermentation of non-digestible carbohydrates and exogenous forms in ameliorating intestinal mucosal immunity of young ruminants. Front Immunol. 2023;14:1291846. 10.3389/fimmu.2023.1291846. 38149240 10.3389/fimmu.2023.1291846 PMC10750390

[3] Malmuthuge N, Griebel PJ, Guan LL. The gut microbiome and its potential role in the development and function of newborn calf gastrointestinal tract. Front Vet Sci. 2015;2:36. 10.3389/fvets.2015.00036. 26664965 10.3389/fvets.2015.00036 PMC4672224

[4] Liang G, Malmuthuge N, Bao H, Stothard P, Griebel PJ, Guan LL. Transcriptome analysis reveals regional and temporal differences in mucosal immune system development in the small intestine of neonatal calves. BMC Genomics. 2016;17:602. 10.1186/s12864-016-2957-y. 10.1186/s12864-016-2957-y PMC4981982 27515123

[5] Sun D, Bian G, Zhang K, Liu N, Yin Y, Hou Y, et al. Early-life ruminal microbiome-derived indole-3-carboxaldehyde and prostaglandin D2 are effective promoters of rumen development. Genome Biol. 2024;25:64. 10.1186/s13059-024-03205-x. 10.1186/s13059-024-03205-x PMC10910749 38438919

[6] Malmuthuge N, Liang G, Guan LL. Regulation of rumen development in neonatal ruminants through microbial metagenomes and host transcriptomes. Genome Biol. 2019;20:172. 10.1186/s13059-019-1786-0. 10.1186/s13059-019-1786-0 PMC6708143 31443695

[7] Alipour MJ, Jalanka J, Pessa-Morikawa T, Kokkonen T, Satokari R, Hynönen U, et al. The composition of the perinatal intestinal microbiota in cattle. Sci Rep. 2018;8:10437. 10.1038/s41598-018-28733-y. 10.1038/s41598-018-28733-y PMC6041309 29993024

[8] Song Y, Malmuthuge N, Steele MA, Guan LL. Shift of hindgut microbiota and microbial short chain pre-weaning. FEMS Microbial Ecol. 2018. 10.1093/femsec/fix179. 10.1093/femsec/fix179 29267960

[9] Li F, Li C, Chen Y, Liu J, Zhang C, Irving B, et al. Host genetics influence the rumen microbiota and heritable rumen microbial features associate with feed efficiency in cattle. Microbiome. 2019;7:92. 10.1186/s40168-019-0699-1. 10.1186/s40168-019-0699-1 PMC6567441 31196178

[10] Guryanova SV, Ovchinnikova TV. Immunomodulatory and allergenic properties of antimicrobial peptides. Int J Mol Sci. 2022;23(5):2499. 10.3390/ijms23052499. 35269641 10.3390/ijms23052499 PMC8910669

[11] Rostoll Cangiano L, Villot C, Amorin-Hegedus R, Malmuthuge N, Gruninger R, Guan LL, et al. *Saccharomyces cerevisiae**boulardii* accelerates intestinal microbiota maturation and is correlated with increased secretory IgA production in neonatal dairy calves. Front Microbiol. 2023;14:1129250. 10.3389/fmicb.2023.1129250. 37795296 10.3389/fmicb.2023.1129250 PMC10546063

[12] Hegyi P, Maléth J, Walters JR, Hofmann AF, Keely SJ. Guts and gall: bile acids in regulation of intestinal epithelial function in health and disease bile acid physiology pathological effects of bile acids. Physiol Rev. 2028;98(4):1983–2023. 10.1152/physrev.00054.2017. 10.1152/physrev.00054.2017 30067158

[13] Shang T, Zhang R, Liu Y, Shi S. Intestinal oxygen and microbiota crosstalk : implications for pathogenesis of gastrointestinal diseases and emerging therapeutic strategies. Gut Pathog. 2025;17:100. 10.1186/s13099-025-00783-4. 41361482 10.1186/s13099-025-00783-4 PMC12683929

[14] Meale SJ, Li SC, Azevedo P, Derakhshani H, DeVries TJ, Plaizier JC, et al. Weaning age influences the severity of gastrointestinal microbiome shifts in dairy calves. Sci Rep. 2017;7:198. 10.1038/s41598-017-00223-7. 10.1038/s41598-017-00223-7 PMC5428063 28298634

[15] Steele MA, Penner GB, Chaucheyras-Durand F, Guan LL. Development and physiology of the rumen and the lower gut: targets for improving gut health. J Dairy Sci. 2016;99(6):4955–66. 10.3168/jds.2015-10351. 26971143 10.3168/jds.2015-10351

[16] Liu M, Lu Y, Xue G, Han L, Jia H, Wang Z, et al. Role of short-chain fatty acids in host physiology. Anim Model Exp Med. 2024;7(5):641–52. 10.1002/ame2.12464. 10.1002/ame2.12464 PMC11528394 38940192

[17] Park J, Kotani T, Konno T, Setiawan J, Kitamura Y. Promotion of intestinal epithelial cell turnover by commensal bacteria : role of short-chain fatty acids. PLoS ONE. 2016;11(5):e0156334. 10.1371/journal.pone.0156334. 27232601 10.1371/journal.pone.0156334 PMC4883796

[18] Zhang K, Xu Y, Yang Y, Guo M, Zhang T, Zong B, et al. Gut microbiota-derived metabolites contribute negatively to hindgut barrier function development at the early weaning goat model. Anim Nutr. 2022;21(10):111–23. 10.1016/j.aninu.2022.04.004. 10.1016/j.aninu.2022.04.004 PMC9136126 35663372

[19] Li C, Wang W, Liu T, Zhang Q, Wang G, Li F, et al. Effect of early weaning on the intestinal microbiota and expression of genes related to barrier function in lambs. Front Microbiol. 2018;9:1431. 10.3389/fmicb.2018.01431. 30013534 10.3389/fmicb.2018.01431 PMC6036172

[20] Brown EM, Kenny DJ, Xavier RJ. Gut microbiota regulation of T Cells during inflammation and autoimmunity. Annu Rev Immunol. 2019;37:599–624. 10.1146/annurev-immunol-042718-041841. 31026411 10.1146/annurev-immunol-042718-041841

[21] Díaz-Garrido N, Badia J, Baldomà L. Microbiota-derived extracellular vesicles in interkingdom communication in the gut. J Extracell Vesicles. 2021;10(13):e12161. 10.1002/jev2.12161. 34738337 10.1002/jev2.12161 PMC8568775

[22] Guo N, Gou N, Shi F, Wang W, Li S, Bi S, et al. Short-chain fatty acids mediate interactions between immune responses and commensal bacteria in high-altitude yaks. Commun Biol. 2026;9:81. 10.1038/s42003-025-09351-7. 10.1038/s42003-025-09351-7 PMC12820265 41390545

[23] Guo W, Van Niekerk JK, Zhou M, Steele MA, Guan LL. Longitudinal assessment revealed the shifts in rumen and colon mucosa-attached microbiota of dairy calves during weaning transition. J Dairy Sci. 2021;104(5):5948–63. 10.3168/jds.2020-19252. 33612210 10.3168/jds.2020-19252

[24] Mirzaei R, Bouzari B, Hosseini-Fard SR, Mazaheri M, Ahmadyousefi Y, Abdi M, et al. Role of microbiota-derived short-chain fatty acids in nervous system disorders. Biomed Pharmacother. 2021;139:111661. 10.1016/j.biopha.2021.111661. 34243604 10.1016/j.biopha.2021.111661

[25] Spiljar M, Merkler D, Trajkovski M. The immune system bridges the gut microbiota with systemic energy homeostasis: focus on TLRs, mucosal barrier, and SCFAs. Front Immunol. 2017;8:1353. 10.3389/fimmu.2017.01353. 29163467 10.3389/fimmu.2017.01353 PMC5670327

[26] Huang SC, He YF, Chen P, Liu KL, Shaukat A. Gut microbiota as a target in the bone health of livestock and poultry : roles of short-chain fatty acids. Anim Dis. 2023;3:23. 10.1186/s44149-023-00089-5.

[27] Huang J, Wang X, Wang Z, Deng L, Wang Y, Tang Y, et al. Cytokine and growth factor reviews extracellular vesicles as a novel mediator of interkingdom communication. Cytokine Growth Factor Rev. 2023;73:173–84. 10.1016/j.cytogfr.2023.08.005. 10.1016/j.cytogfr.2023.08.005 37634980

[28] Yoo JY, Groer M, Dutra SVO, Sarkar A, McSkimming DI. Gut microbiota and immune system interactions. Microorganisms. 2020;8(10):1587. 10.3390/microorganisms8101587. 33076307 10.3390/microorganisms8101587 PMC7602490

[29] Chigozie VU, Enyi EO, Akwari AA, Esimone CO. Host-microbe interactions at barrier tissues and immunomodulation : a scoping review. Discov Bacteria. 2025;2:26. 10.1007/s44351-025-00037-3.

[30] Amoroso C, Perillo F, Strati F, Fantini MC, Caprioli F, Facciotti F. The role of gut microbiota biomodulators on mucosal immunity and intestinal inflammation. Cells. 2020;9(5):1234. 10.3390/cells9051234. 32429359 10.3390/cells9051234 PMC7291275

[31] Diddeniya G, Ghaffari MH, Hernandez-sanabria E, Guan LL. Invited review: impact of maternal health and nutrition on the microbiome and immune development of neonatal calves. J Dairy Sci. 2024;107(10):7504–19. 10.3168/jds.2024-24835. 38825126 10.3168/jds.2024-24835

[32] Liu S, Yang L, Zhang Y, Chen H, Li X, Xu Z, et al. Review of yeast culture concerning the interactions between gut microbiota and young ruminant animals. Front Vet Sci. 2024;11:1335765. 10.3389/fvets.2024.1335765. 10.3389/fvets.2024.1335765 PMC10940410 38496306

[33] Liu Y, Wang J, Wu C. Modulation of gut microbiota and immune system by probiotics, pre-biotics, and postbiotics. Front Nutr. 2022;8:634897. 10.3389/fnut.2021.634897. 35047537 10.3389/fnut.2021.634897 PMC8761849

[34] Lin L, Xie F, Sun D, Liu J, Zhu W, Mao S. Ruminal microbiome-host crosstalk stimulates the development of the ruminal epithelium in a lamb model. Microbiome. 2019;7(1):83. 10.1186/s40168-019-0701-y. 31159860 10.1186/s40168-019-0701-y PMC6547527

[35] Wu Y, Wang L, Luo R, Chen H, Nie C, Niu J, et al. Effect of a multispecies probiotic mixture on the growth and incidence of diarrhea, immune function, and fecal microbiota of pre-weaning dairy calves. Front Microbiol. 2021;12:681014. 10.3389/fmicb.2021.681014. 34335503 10.3389/fmicb.2021.681014 PMC8318002

[36] Wang Y, Liu W, Li A, Qubi W, Gong C, Li X, et al. Changes in the growth performance, serum biochemistry, rumen fermentation, rumen microbiota community, and intestinal development in weaned goats during rumen-protected methionine treatment. Front Vet Sci. 2024;11:1482235. 10.3389/fvets.2024.1482235. 39582883 10.3389/fvets.2024.1482235 PMC11582046

[37] Arshad MA, Hassan F, Rehman MS, Huws SA, Cheng Y, Din AU. Gut microbiome colonization and development in neonatal ruminants: strategies, prospects, and opportunities. Anim Nutr. 2021;7(3):883–95. 10.1016/j.aninu.2021.03.004. 34632119 10.1016/j.aninu.2021.03.004 PMC8484983

[38] Godlewska U, Bulanda E, Wypych TP. Bile acids in immunity: bidirectional mediators between the host and the microbiota. Front Immunol. 2022;13:949033. 10.3389/fimmu.2022.949033. 36052074 10.3389/fimmu.2022.949033 PMC9425027

[39] Sozinov AS, Anikhovskaya IA, Zhdanov RI, Markelova MM, Morozov SG. Intestinal microbiota as a fundamental basis for homeostasis, general pathology and aging. Kazan Med Zh. 2024;105(6):987–93. 10.17816/KMJ633598.

[40] Zhang Y, Choi SH, Nogoy KM, Liang S. Review: the development of the gastrointestinal tract microbiota and intervention in neonatal ruminants. Animal. 2021;15(8):100316. 10.1016/j.animal.2021.100316. 34293582 10.1016/j.animal.2021.100316

[41] Li K, Shi B, Na R. The colonization of Rumen microbiota and intervention in pre-weaned ruminants. Animals. 2023;13(6):994. 10.3390/ani13060994. 36978535 10.3390/ani13060994 PMC10044309

[42] Yang F, Wang A, Zeng X, Hou C, Liu H, Qiao S. *Lactobacillus reuteri* I5007 modulates tight junction protein expression in IPEC-J2 cells with LPS stimulation and in newborn piglets under normal conditions. BMC Microbiol. 2015;15:32. 10.1186/s12866-015-0372-1. 25888437 10.1186/s12866-015-0372-1 PMC4350629

[43] Ling X, Linglong P, Weixia D, Hong W. Protective effects of *Bifidobacterium* on intestinal barrier function in LPS-induced enterocyte barrier injury of Caco-2 monolayers and in a rat NEC model. PLoS ONE. 2016;11(8):e0161635. 10.1371/journal.pone.0161635. 10.1371/journal.pone.0161635 PMC4995054 27551722

[44] Martín R, Laval L, Chain F, Miquel S, Natividad J, Cherbuy C, et al. CNCM-I2494 *Bifidobacterium animalis *ssp*. lactis* CNCM-I2494 restores gut barrier permeability in chronically low-grade inflamed mice. Front Microbiol. 2016;7:608. 10.3389/fmicb.2016.00608. 10.3389/fmicb.2016.00608 PMC4858658 27199937

[45] Pontarollo G, Kollar B, Mann A, Khuu MP, Kiouptsi K, Bayer F, et al. Commensal bacteria weaken the intestinal barrier by suppressing epithelial neuropilin-1 and Hedgehog signaling. Nat Metab. 2023;5(7):1174–87. 10.1038/s42255-023-00828-5. 37414930 10.1038/s42255-023-00828-5 PMC10365997

[46] Al-sadi R, Dharmaprakash V, Nighot P, Guo S, Nighot M, Do T. *Bifidobacterium bifidum* enhances the intestinal epithelial tight junction barrier and protects against intestinal inflammation by targeting the toll-like receptor-2 pathway in an NF-κB-independent manner. Int J Mol Sci. 2021;22(15):8070. 10.3390/ijms22158070. 10.3390/ijms22158070 PMC8347470 34360835

[47] Beaumont M, Paës C, Mussard E, Knudsen C, Cauquil L, Aymard P, et al. Gut microbiota derived metabolites contribute to intestinal barrier maturation at the suckling-to-weaning transition. Gut Microbes. 2020;11(5):1268–86. 10.1080/19490976.2020.1747335. 32352849 10.1080/19490976.2020.1747335 PMC7524271

[48] Gieryńska M, Szulc-Dąbrowska L, Struzik J, Mielcarska MB, Gregorczyk-Zboroch KP. Integrity of the intestinal barrier: the involvement of epithelial cells and microbiota—a mutual relationship. Animals (Basel). 2022;12(2):145. 10.3390/ani12020145. 35049768 10.3390/ani12020145 PMC8772550

[49] Yin J, Zhou C, Yang K, Ren Y, Qiu Y, Xu P, et al. Mutual regulation between butyrate and hypoxia-inducible factor-1α in epithelial cell promotes expression of tight junction proteins. Cell Biol Int. 2020;44(6):1405–14. 10.1002/cbin.11336. 10.1002/cbin.11336 32129567

[50] Gao P, Pang S, Wang Q, Tang Y, Li Q, Zhang W, et al. *Clostridium butyricum* supplementation reduces diarrhea in preweaning calves by modulating fecal short-chain fatty acids and gut microbiota. Microorganisms. 2025;13(9):1993. 10.3390/microorganisms13091993. 41011325 10.3390/microorganisms13091993 PMC12471609

[51] Wu D, Zhang Z, Song Q, Jia Y, Qi J. Modulating gastrointestinal microbiota in preweaning dairy calves : dose-dependent effects of milk-based sodium butyrate supplementation. Microorganisms. 2024;12(2):333. 10.3390/microorganisms12020333. 38399737 10.3390/microorganisms12020333 PMC10893347

[52] Park M, Choi YY, Lee Y, Cho M. Single radiation exposure induces gut microbiota dysbiosis and decreases short-chain fatty acid metabolism and intestinal barrier integrity in mice. Front Cell Infect Microbiol. 2025;15:1654976. 10.3389/fcimb.2025.1654976. 41040983 10.3389/fcimb.2025.1654976 PMC12484129

[53] Weström B, Arévalo Sureda E, Pierzynowska K, Pierzynowski SG, Pérez-Cano FJ. The immature gut barrier and its importance in establishing immunity in newborn mammals. Front Immunol. 2020;11:1153. 10.3389/fimmu.2020.01153. 32582216 10.3389/fimmu.2020.01153 PMC7296122

[54] Dmytriv TR, Storey KB, Lushchak VI. Intestinal barrier permeability: the influence of gut microbiota, nutrition, and exercise. Front Physiol. 2024;15:1380713. 10.3389/fphys.2024.1380713. 39040079 10.3389/fphys.2024.1380713 PMC11260943

[55] Upadhaya S. The impact of weaning stress on gut health and the mechanistic aspects of several feed additives contributing to improved gut health function in weanling piglets. Animals (Basel). 2021;11(8):2418. 10.3390/ani11082418. 34438875 10.3390/ani11082418 PMC8388735

[56] Joshi H, Bernard B, Lemley C, Wang Z, Fan P. Unveiling the rumen-microbiome-brain circuit : a unique dimension of gut-brain axis in ruminants. J Anim Sci Biotechnol. 2025;16:159. 10.1186/s40104-025-01289-4. 10.1186/s40104-025-01289-4 PMC12659478 41310771

[57] Ma J, Liu M, Xu J, Liu B, Cui Y, Shi Y. Fecal microbiota transplantation mitigates lipopolysaccharide - induced oxidative stress in weaned piglets by modulating gut microbiota and enhancing riboflavin metabolism. J Anim Sci Biotechnol. 2026;17:9. 10.1186/s40104-025-01330-6. 10.1186/s40104-025-01330-6 PMC12809982 41545905

[58] Ma X, Zhang Y, Xu T, Qian M, Yang Z, Zhan X. Early-life intervention using exogenous fecal microbiota alleviates gut injury and reduces inflammation caused by weaning stress in piglets. Front Microbiol. 2021;12:671683. 10.3389/fmicb.2021.671683. 34177852 10.3389/fmicb.2021.671683 PMC8222923

[59] St-pierre B, Yair J, Palencia P, Samuel RS. Impact of early weaning on development of the swine gut microbiome. Microorganisms. 2023;11(7):1753. 10.3390/microorganisms11071753. 37512925 10.3390/microorganisms11071753 PMC10385335

[60] Liu M, Ma J, Xu J, Huangfu W, Zhang Y, Ali Q. Fecal microbiota transplantation alleviates intestinal inflammatory diarrhea caused by oxidative stress and pyroptosis via reducing gut microbiota-derived lipopolysaccharides. Int J Biol Macromol. 2024;261(1):129696. 10.1016/j.ijbiomac.2024.129696. 38280701 10.1016/j.ijbiomac.2024.129696

[61] Li S, Mu R, Zhang Y, Wang S, Wright ADG, Si H, et al. Dynamics of intestinal mucosa microbiota in juvenile sika deer during early growth. Int J Mol Sci. 2025;26(3):892. 10.3390/ijms26030892. 39940663 10.3390/ijms26030892 PMC11817005

[62] Lach G, Schellekens H, Dinan TG, Cryan JF. Anxiety, depression, and the microbiome : a role for gut peptides. Neurotherapeutics. 2018;15(1):36–59. 10.1007/s13311-017-0585-0. 29134359 10.1007/s13311-017-0585-0 PMC5794698

[63] Izuddin WI, Loh TC, Foo HL, Samsudin AA, Humam AM. Postbiotic L. plantarum RG14 improves ruminal epithelium growth, immune status and upregulates the intestinal barrier function in post-weaning lambs. Sci Rep. 2019;9:9938. 10.1038/s41598-019-46076-0. 10.1038/s41598-019-46076-0 PMC6616331 31289291

[64] Du H, Li K, Guo W, Na M, Zhang J, Na R. Maternal roughage sources influence the gastrointestinal development of goat kids by modulating the colonization of gastrointestinal microbiota. Animals. 2025;15(3):393. 10.3390/ani15030393. 39943163 10.3390/ani15030393 PMC11815875

[65] Hu QY, Man JJ, Luo J, Cheng F, Yang M, Lin G, et al. Early-life supplementation with mannan-rich fraction to regulate rumen microbiota , gut health , immunity , and growth performance in dairy goat kids. J Dairy Sci. 2024;107(11):9322–33. 10.3168/jds.2024-24903. 39004122 10.3168/jds.2024-24903

[66] Pan X, Li Z, Li B, Zhao C, Wang Y, Chen Y, et al. Dynamics of rumen gene expression, microbiome colonization, and their interplay in goats. BMC Genomics. 2021;22:288. 10.1186/s12864-021-07595-1. 33882826 10.1186/s12864-021-07595-1 PMC8059226

[67] Wickramasinghe J, Anderson CJ, Kaya CA, Gorden PJ, Ribeiro FRB, Dohms J, et al. Evaluating ruminal and small intestinal morphology and microbiota composition of calves fed a Macleaya cordata extract preparation. Animals. 2023;13(1):54. 10.3390/ani13010054. 10.3390/ani13010054 PMC9817497 36611664

[68] Choudhury R, Middelkoop A, de Souza JG, van Veen LA, Gerrits WJJ, Kemp B, et al. Impact of early-life feeding on local intestinal microbiota and digestive system development in piglets. Sci Rep. 2021;11:4213. 10.1038/s41598-021-83756-2. 10.1038/s41598-021-83756-2 PMC7892833 33603087

[69] Wang Q, Wang Y, Wang X, Dai C, Tang W, Li J, et al. Effects of dietary energy levels on rumen fermentation, microbiota, and gastrointestinal morphology in growing ewes. Food Sci Nutr. 2020;8(12):6621–32. 10.1002/fsn3.1955. 33312546 10.1002/fsn3.1955 PMC7723210

[70] Zhou J, Ren Y, Wen X, Yue S, Zou H, Jiang Y, et al. Comparison of coated and uncoated trace elements on growth performance, apparent digestibility, intestinal development and microbial diversity in growing sheep. Front Microbiol. 2022;13:1080182. 10.3389/fmicb.2022. 36605519 10.3389/fmicb.2022.1080182 PMC9808050

[71] Xie J, Li L, Dai T, Qi X, Wang Y, Zheng T, et al. Short-chain fatty scids produced by Ruminococcaceae mediate α-linolenic acid promote intestinal stem cells proliferation. Mol Nutr Food Res. 2022;66(1):e2100408. 10.1002/mnfr.202100408. 34708542 10.1002/mnfr.202100408

[72] Zhong H, Yu W, Wang M, Lin B, Sun X, Zheng N, et al. Sodium butyrate promotes gastrointestinal development of preweaning bull calves via inhibiting inflammation, balancing nutrient metabolism, and optimizing microbial community functions. Anim Nutr. 2023;14:88–100. 10.1016/j.aninu.2023.04.004. 37388163 10.1016/j.aninu.2023.04.004 PMC10300058

[73] Murayama K, Fukui T, Isobe N, Oba M, Sugino T. Effects of tributyrin supplementation in calf starter on growth, blood parameters and development of rumen and small intestine in Holstein calves fed milk replacers with different fatty acid profile. Anim Feed Sci Technol. 2024;308:115883. 10.1016/j.anifeedsci.2024.115883.

[74] Lu L, Yu R, Du Z, Bing H, Lin G, Hui S. Ginseng promotes the function of intestinal stem cells through the Wnt/β-catenin signaling pathway in D-galactose-induced aging mice. Exp Gerontol. 2024;185:112351. 10.1016/j.exger.2023.112351. 10.1016/j.exger.2023.112351 38135257

[75] Liu Y, Yan T, Ren Z, Yang X. Age-associated changes in caecal microbiome and their apparent correlations with growth performances of layer pullets. Anim Nutr. 2021;7(3):841–8. 10.1016/j.aninu.2020.11.019. 34466688 10.1016/j.aninu.2020.11.019 PMC8379648

[76] Paeslack N, Reinhardt C. The impact of gut microbiota on vascularization of the small intestine. Microbiota and Host. 2024;2:1. 10.1530/MAH-23-0021.

[77] Bayer F, Dremova O, Khuu MP, Mammadova K, Pontarollo G, Kiouptsi K, et al. The interplay between nutrition, innate immunity, and the commensal microbiota in adaptive intestinal morphogenesis. Nutrients. 2021;13(7):2198. 10.3390/nu13072198. 34206809 10.3390/nu13072198 PMC8308283

[78] Mishima Y, Oka A, Liu B, Herzog JW, Eun CS, Fan TJ, et al. Microbiota maintain colonic homeostasis by activating TLR2/MyD88/PI3K signaling in IL-10-producing regulatory B cells. J Clin Invest. 2019;129(9):3702–16. 10.1172/JCI93820. 31211700 10.1172/JCI93820 PMC6715367

[79] Sommer F, Bäckhed F. Know your neighbor: microbiota and host epithelial cells interact locally to control intestinal function and physiology. BioEssays. 2016;38(5):455–64. 10.1002/bies.201500151. 26990415 10.1002/bies.201500151

[80] Okumura R, Takeda K. Roles of intestinal epithelial cells in the maintenance of gut homeostasis. Exp Mol Med. 2017;49(5):e338–48. 10.1038/emm.2017.20. 28546564 10.1038/emm.2017.20 PMC5454438

[81] Kayama H, Okumura R, Takeda K. Interaction between the microbiota, epithelia, and immune cells in the intestine. Annu Rev Immunol. 2020;38:23–48. 10.1146/annurev-immunol-070119-115104. 32340570 10.1146/annurev-immunol-070119-115104

[82] Soderholm AT, Pedicord VA. Intestinal epithelial cells: at the interface of the microbiota and mucosal immunity. Immunology. 2019;158(4):267–80. 10.1111/imm.13117. 31509239 10.1111/imm.13117 PMC6856932

[83] Yao Y, Shang W, Bao L, Peng Z, Wu C. Epithelial-immune cell crosstalk for intestinal barrier homeostasis. Eur J Immunol. 2024;54(6):e2350631. 10.1002/eji.202350631. 38556632 10.1002/eji.202350631

[84] Martens EC, Neumann M, Desai MS. Interactions of commensal and pathogenic microorganisms with the intestinal mucosal barrier. Nat Rev Microbiol. 2018;16(8):457–70. 10.1038/s41579-018-0036-x. 29904082 10.1038/s41579-018-0036-x

[85] Yang S, Liu H, Liu Y. Advances in intestinal epithelium and gut microbiota interaction. Front Microbiol. 2025;16:1499202. 10.3389/fmicb.2025.1499202. 40104591 10.3389/fmicb.2025.1499202 PMC11914147

[86] Amimo JO, Raev SA, Chepngeno J, Mainga AO, Guo Y, Saif L, et al. Rotavirus interactions with host intestinal epithelial cells. Front Immunol. 2021;12:793841. 10.3389/fimmu.2021.793841. 35003114 10.3389/fimmu.2021.793841 PMC8727603

[87] Didriksen BJ, Eshleman EM, Alenghat T. Epithelial regulation of microbiota-immune cell dynamics. Mucosal Immunol. 2024;17(2):303–13. 10.1016/j.mucimm.2024.02.008. 38428738 10.1016/j.mucimm.2024.02.008 PMC11412483

[88] Malmuthuge N, Guan LL. Understanding the gut microbiome of dairy calves: opportunities to improve early-life gut health. J Dairy Sci. 2017;100(7):5996–6005. 10.3168/jds.2016-12239. 28501408 10.3168/jds.2016-12239

[89] Mu R, Li S, Zhang Y, Li Y, Zhu Y, Zhao F, et al. Microbiota and metabolite profiles in the feces of juvenile sika deer (*Cervus nippon*) from birth to weaning. Animals (Basel). 2024;14(3):432. 10.3390/ani14030432. 10.3390/ani14030432 PMC10854736 38338075

[90] Guo J, Li P, Zhang K, Zhang L, Wang X, Li L, et al. Distinct stage changes in early-life colonization and acquisition of the gut microbiota and its correlations with volatile fatty acids in goat kids. Front Microbiol. 2020;11:584742. 10.3389/fmicb.2020.584742. 33162961 10.3389/fmicb.2020.584742 PMC7581860

[91] Bi Y, Cox MS, Zhang F, Suen G, Zhang N, Tu Y, et al. Feeding modes shape the acquisition and structure of the initial gut microbiota in newborn lambs. Environ Microbiol. 2019;21(7):2333–46. 10.1111/1462-2920.14614. 30938032 10.1111/1462-2920.14614 PMC6849743

[92] Sanjorjo RA, Tseten T, Kang M, Kwon M. In pursuit of understanding the Rumen microbiome. Fermentation. 2023;9:114. 10.3390/fermentation9020114.

[93] Balouei F, Stefanon B, Sgorlon S, Sandri M. Factors affecting gut microbiota of puppies from birth to weaning. Animals Basel. 2023;13(4):578. 10.3390/ani13040578. 36830365 10.3390/ani13040578 PMC9951692

[94] Mach N, Monié-ibanes M, Sikht FZ, Hygonenq M, Pot G, Robert H, et al. Decoding the dynamics of calves ’ respiratory and gut microbiota : exploring stability , resistance , and individual patterns. Anim Microbiome. 2025;7:126. 10.1186/s42523-025-00494-w. 41413607 10.1186/s42523-025-00494-w PMC12713259

[95] Meale SJ, Chaucheyras-Durand F, Berends H, Guan LL, Steele MA. From pre- to postweaning: transformation of the young calf’s gastrointestinal tract. J Dairy Sci. 2017;100(7):5984–95. 10.3168/jds.2016-12474. 28527800 10.3168/jds.2016-12474

[96] Wang W, Li C, Li F, Wang X, Zhang X, Liu T, et al. Effects of early feeding on the host rumen transcriptome and bacterial diversity in lambs. Sci Rep. 2016;6:32479. 10.1038/srep32479. 27576848 10.1038/srep32479 PMC5006043

[97] Dill-McFarland KA, Weimer PJ, Breaker JD, Suen G. Diet influences early microbiota development in dairy calves without long-term impacts on milk production. Appl Environ Microbiol. 2019;85(2):e02141-18. 10.1128/AEM.02141-18. 30367001 10.1128/AEM.02141-18 PMC6328763

[98] Huuki H, Vilkki J, Vanhatalo A, Tapio I. Fecal microbiota colonization dynamics in dairy heifers associated with early-life rumen microbiota modulation and gut health. Front Microbiol. 2024;15:1353874. 10.3389/fmicb.2024.1353874. 38505558 10.3389/fmicb.2024.1353874 PMC10949896

[99] Guo J, Han X, You Y, Huang W, Zhan J. IgA dysfunction induced by the early-lifetime disruption of gut microbiota aggravates diet – induced metabolic syndrome in mice. 2020. 10.21203/rs.3.rs-40336/v1.

[100] Alawneh JI, Ramay H, Olchowy T, Allavena R, Soust M, Al Jassim R. Effect of a lactobacilli-based direct-fed microbial product on gut microbiota and gastrointestinal morphological changes. Animals. 2024;14:693. 10.3390/ani14050693. 38473078 10.3390/ani14050693 PMC10931233

[101] Pan Z, Ma T, Steele M, Guan LL. Varied microbial community assembly and specialization patterns driven by early life microbiome perturbation and modulation in young ruminants. ISME Commun. 2024;4(1):ycae044. 10.1093/ismeco/ycae044. 38650709 10.1093/ismeco/ycae044 PMC11033733

[102] Fan P, Bian B, Teng L, Nelson CD, Driver J, Elzo MA, et al. Host genetic effects upon the early gut microbiota in a bovine model with graduated spectrum of genetic variation. ISME J. 2020;14(1):302–17. 10.1038/s41396-019-0529-2. 31624342 10.1038/s41396-019-0529-2 PMC6908690

[103] Fan P, Nelson CD, Driver JD, Elzo MA, Peñagaricano F, Jeong KC. Host genetics exerts lifelong effects upon hindgut microbiota and its association with bovine growth and immunity. ISME J. 2021;15(8):2306–21. 10.1038/s41396-021-00925-x. 33649551 10.1038/s41396-021-00925-x PMC8319427

[104] Déru V, Bouquet A, Zemb O, Blanchet B, De Almeida ML, Cauquil L, et al. Genetic relationships between efficiency traits and gut microbiota traits in growing pigs being fed with a conventional or a high-fiber diet. J Anim Sci. 2022;100(6):skac183. 10.1093/jas/skac183. 10.1093/jas/skac183 PMC9194801 35579995

[105] Aliakbari A, Zemb O, Cauquil L, Barilly C, Billon Y, Gilbert H. Microbiability and microbiome-wide association analyses of feed efficiency and performance traits in pigs. Genet Sel Evol. 2022;54(1):29. 10.1186/s12711-022-00717-7. 35468740 10.1186/s12711-022-00717-7 PMC9036775

[106] Crespo-Piazuelo D, Migura-Garcia L, Estellé J, Criado-Mesas L, Revilla M, Castelló A, et al. Association between the pig genome and its gut microbiota composition. Sci Rep. 2019;9:8791. 10.1038/s41598-019-45066-6. 10.1038/s41598-019-45066-6 PMC6584621 31217427

[107] Larzul C, Estellé J, Borey M, Blanc F, Lemonnier G, Billon Y, et al. Driving gut microbiota enterotypes through host genetics. Microbiome. 2024;12:116. 10.1186/s40168-024-01827-8. 10.1186/s40168-024-01827-8 PMC11214205 38943206

[108] Abbas W, Howard JT, Paz HA, Hales KE, Wells JE, Kuehn LA, et al. Influence of host genetics in shaping the rumen bacterial community in beef cattle. Sci Rep. 2020;10:15101. 10.1038/s41598-020-72011-9. 10.1038/s41598-020-72011-9 PMC7493918 32934296

[109] Bessegatto JA, Lisbôa JAN, Santos BP, Curti JM, Montemor C, Alfieri AA, et al. Fecal microbial communities of nellore and crossbred beef calves raised at pasture. Animals. 2024;14(10):1447. 10.3390/ani14101447. 38791664 10.3390/ani14101447 PMC11117347

[110] Huang J, Li Y. Rumen methanogen and protozoal communities of Tibetan sheep and Gansu Alpine Finewool sheep grazing on the Qinghai–Tibetan Plateau, China. BMC Microbiol. 2018;18:212. 10.1186/s12866-018-1351-0. 10.1186/s12866-018-1351-0 PMC6293568 30545295

[111] Heras-Molina A, Estellé J, Vázquez-Gómez M, López-García A, Pesantez-Pacheco JL, Astiz S, et al. The impact of host genetics on porcine gut microbiota composition excluding maternal and postnatal environmental influences. PLoS ONE. 2024;19(12):e0315199. 10.1371/journal.pone.0315199. 39652543 10.1371/journal.pone.0315199 PMC11627362

[112] Kim SH, Sung HG. Effects of different fiber substrates on in vitro rumen fermentation characteristics and rumen microbial community in Korean native goats and hanwoo steers. Fermentation. 2022; 8(11):611. 10.3390/fermentation8110611.

[113] Holman DB, Gzyl KE. A meta-analysis of the bovine gastrointestinal tract microbiota. FEMS Microbiol Ecol. 2019;95(6):fiz072. 10.1093/femsec/fiz072. 31116403 10.1093/femsec/fiz072

[114] Qiu X, Qin X, Chen L, Chen Z, Hao R, Zhang S, et al. Serum biochemical parameters, rumen fermentation, and rumen bacterial communities are partly driven by the breed and sex of cattle when fed high-grain diet. Microorganisms. 2022;10(2):323. 10.3390/microorganisms10020323. 35208778 10.3390/microorganisms10020323 PMC8878564

[115] Su L, Guo J, Shi W, Tong W, Li X, Yang B, et al. Metagenomic analysis reveals the composition and function of intestinal microbiota were affected by different sampling methods and digestive tract segments in monogastric and polygastric animals. BMC Microbiol. 2023;24:530. 10.1186/s12866-024-03696-5.

[116] Du Y, Gao Y, Hu M, Hou J, Yang L, Wang X, et al. Colonization and development of the gut microbiome in calves. J Anim Sci Biotechnol. 2023;14:46. 10.1186/s40104-023-00856-x. 10.1186/s40104-023-00856-x PMC10082981 37031166

[117] Okpara MO, Ozuo UK. Understanding the impact of diet, host genetics and early life interventions on rumen microbiome. Niger J Anim Prod. 2024:1847–50. 10.51791/njap.vi.7354.

[118] Mani S, Aiyegoro OA, Adeleke MA. Association between host genetics of sheep and the rumen microbial composition. Trop Anim Health Prod. 2020;54(2):109. 10.1007/s11250-022-03057-2. 10.1007/s11250-022-03057-2 35192073

[119] Wang W, Zhang Y, Zhang X, Li C, Yuan L, Zhang D, et al. Heritability and recursive influence of host genetics on the rumen microbiota drive body weight variance in male Hu sheep lambs. Microbiome. 2023;11:197. 10.1186/s40168-023-01642-7. 10.1186/s40168-023-01642-7 PMC10463499 37644504

[120] Sun G, Xia T, Wei Q, Dong Y, Zhao C, Yang X, et al. Analysis of gut microbiota in three species belonging to different genera (*Hemitragus*, *Pseudois*, and *Ovis*) from the subfamily Caprinae in the absence of environmental variance. Ecol Evol. 2021;11(17):12129–40. 10.1002/ece3.7976. 10.1002/ece3.7976 PMC8427585 34522365

[121] Martinez Boggio G, Meynadier A, Buitenhuis AJ, Marie-Etancelin C. Host genetic control on rumen microbiota and its impact on dairy traits in sheep. Genet Sel Evol. 2022;54(1):77. 10.1186/s12711-022-00769-9. 10.1186/s12711-022-00769-9 PMC9694848 36434501

[122] Wang X, Zhang Z, Wang X, Bao Q, Wang R, Duan Z. The impact of host genotype, intestinal sites and probiotics supplementation on the gut microbiota composition and diversity in sheep. Biology (Basel). 2021;10(8):769. 10.3390/biology10080769. 34440001 10.3390/biology10080769 PMC8389637

[123] Jiang X, Xu HJ, Cui ZQ, Zhang YG. Effects of supplementation with *Lactobacillus plantarum* 299v on the performance, blood metabolites, rumen fermentation and bacterial communities of preweaning calves. Livest Sci. 2020. 10.1016/j.livsci.2020.104120.

[124] Gonzalez-Recio O, Zubiria I, García-Rodríguez A, Hurtado A, Atxaerandio R. Short communication: signs of host genetic regulation in the microbiome composition in 2 dairy breeds: Holstein and Brown Swiss. J Dairy Sci. 2018;101(3):2285–92. 10.3168/jds.2017-13179. 29274973 10.3168/jds.2017-13179

[125] Paz HA, Anderson CL, Muller MJ, Kononoff PJ, Fernando SC. Rumen bacterial community composition in Holstein and Jersey cows is different under same dietary condition and is not affected by sampling method. Front Microbiol. 2016;7:1206. 10.3389/fmicb.2016.01206. 27536291 10.3389/fmicb.2016.01206 PMC4971436

[126] Liang Z, Zhang J, Du M, Ahmad AA, Wang S, Zheng J, et al. Age-dependent changes of hindgut microbiota succession and metabolic function of Mongolian cattle in the semi-arid rangelands. Front Microbiol. 2022;13:957341. 10.3389/fmicb.2022.957341. 35935190 10.3389/fmicb.2022.957341 PMC9354825

[127] Li B, Yin W, Lei M, Wang X, Yang Y, Zhang C, et al. Exploring the digesta- and mucosa-associated microbial community dynamics in the rumen and hindgut of goats from birth to adult. Front Microbiol. 2023;14:1190348. 10.3389/fmicb.2023.1190348. 37396393 10.3389/fmicb.2023.1190348 PMC10311480

[128] Li Z, Wang X, Zhang T, Si H, Nan W, Xu C, et al. The development of microbiota and metabolome in small intestine of sika deer (*Cervus nippon*) from birth to weaning. Front Microbiol. 2018;9:4. 10.3389/fmicb.2018.00004. 29410651 10.3389/fmicb.2018.00004 PMC5787063

[129] Wu Q, Zeng D, Niu Z, Yang X, Liu Y, Yin F, et al. Investigations of morphological development, digestive enzymes and microbiome of the gastrointestinal tract in goat kids from birth to adulthood. J Appl Anim Res. 2024;52(1):2295456. 10.1080/09712119.2023.2295456.

[130] Guo CY, Ji SK, Yan H, Wang YJ, Liu JJ, Cao ZJ, et al. Dynamic change of the gastrointestinal bacterial ecology in cows from birth to adulthood. Microbiologyopen. 2020;9(11):e1119. 10.1002/mbo3.1119. 33034165 10.1002/mbo3.1119 PMC7658451

[131] Zhuang Y, Chai J, Cui K, Bi Y, Diao Q, Huang W, et al. Longitudinal investigation of the gut microbiota in goat kids from birth to postweaning. Microorganisms. 2020;8(8):1111. 10.3390/microorganisms8081111. 32722119 10.3390/microorganisms8081111 PMC7463816

[132] Amin N, Schwarzkopf S, Tröscher-Mußotter J, Camarinha-Silva A, Dänicke S, Huber K, et al. Host metabolome and faecal microbiome shows potential interactions impacted by age and weaning times in calves. Anim Microbiome. 2023;5:12. 10.1186/s42523-023-00233-z. 10.1186/s42523-023-00233-z PMC9926800 36788596

[133] Woodruff KL, Hummel GL, Austin KJ, Lake SL, Cunningham-Hollinger HC. Calf rumen microbiome from birth to weaning and shared microbial properties to the maternal rumen microbiome. J Anim Sci. 2022;100(10):skac264. 10.1093/jas/skac264. 10.1093/jas/skac264 PMC9576027 35986918

[134] Urrutia‑Angulo L, Ocejo M, Yergaliyev T, et al. Exploring colostrum microbiota and its influence on early calf gut microbiota development using full-length 16S rRNA gene metabarcoding. Sci Rep. 2025;15:36350. 10.1038/s41598-025-20111-9. 41107300 10.1038/s41598-025-20111-9 PMC12534416

[135] Lyons T, Jahns H, Brady J, O’Hara E, Waters SM, Kenny D, et al. Integrated analyses of the microbiological, immunological and ontological transitions in the calf ileum during early life. Sci Rep. 2020;10(1):21264. 10.1038/s41598-020-77907-0. 33277514 10.1038/s41598-020-77907-0 PMC7718239

[136] Kim E, Lee S, Kim T, Lee H, Atikur RM, Gu B, et al. Dynamic changes in fecal microbial communities of neonatal dairy calves by aging and diarrhea. Animals (Basel). 2021;11(4):1113. 10.3390/ani11041113. 33924525 10.3390/ani11041113 PMC8070554

[137] Zhao L, Liu X, Gomez NA, Gao Y, Son JS, Chae SA, et al. Stage ‑ specific nutritional management and developmental programming to optimize meat production. J Anim Sci Biotechnol. 2023;14:2. 10.1186/s40104-022-00805-0. 10.1186/s40104-022-00805-0 PMC9809060 36597116

[138] Bush SJ, McCulloch MEB, Muriuki C, Salavati M, Davis GM, Farquhar IL, et al. Comprehensive transcriptional profiling of the gastrointestinal tract of ruminants from birth to adulthood reveals strong developmental stage specific gene expression. G3 (Bethesda). 2019;9(2):359–73. 10.1534/g3.118.200810. 30530642 10.1534/g3.118.200810 PMC6385975

[139] Abdelsattar MM, Zhuang Y, Cui K, Bi Y, Haridy M, Zhang N. Longitudinal investigations of anatomical and morphological development of the gastrointestinal tract in goats from colostrum to postweaning. J Dairy Sci. 2022; 105(3):2597-2611. 10.3168/jds.2021-21056. 10.3168/jds.2021-21056 35086701

[140] Pokhrel B, Tan Z, Jiang H, Sciences A, Tech V. Identification of transcriptional regulators and signaling pathways mediating postnatal rumen growth and functional maturation in cattle. J Anim Sci. 2025;103:367. 10.1093/jas/skae367. 10.1093/jas/skae367 PMC11781194 39656757

[141] Zou X, Liu G, Meng F, Hong L, Li Y. Exploring the rumen and cecum microbial community from fetus to adulthood in goat.Animals (Basel). 2020;10(9):1639. 10.3390/ani10091639. 10.3390/ani10091639 PMC7552217 32932976

[142] Sfulcini M, Lopreiato V, Piccioli-cappelli F, Patrone V, Bisaschi M, Yoon I, et al. *Saccharomyces**cerevisiae* fermentation-derived postbiotics supplementation to dairy calves: effects on growth, metabolism, immune status, and preliminary first-lactation outcomes. Animals (Basel). 2025;15(18):2728. 10.3390/ani15182728. 41007973 10.3390/ani15182728 PMC12466413

[143] Huang Y, Wang G, Li C, Wang W, Zhang X, Wang X, et al. Periodical changes of feces microbiota and its relationship with nutrient digestibility in early lambs. Animals (Basel). 2022;12(14):1770. 10.3390/ani12141770. 35883317 10.3390/ani12141770 PMC9311505

[144] Dias J, Inácio Marcondes M, Motta de Souza S, Cardoso da Mata Silva B, Fontes Noronha M, Tassinari Resende R, et al. Bacterial community dynamics across the gastrointestinal tracts of dairy calves during preweaning development. Appl Envirn Microbiol. 2018; 84(9):e02675–17. 10.1128/AEM.02675-17. 10.1128/AEM.02675-17 PMC5930334 29475865

[145] Jiao J, Huang J, Zhou C, Tan Z. Taxonomic identification of ruminal epithelial bacterial diversity during rumen development in goats. Appl Environ Microbiol. 2015;81(10):3502–9. 10.1128/AEM.00203-15. 25769827 10.1128/AEM.00203-15 PMC4407235

[146] Wang S, Chai J, Zhao G, Zhang N, Cui K, Bi Y, et al. The temporal dynamics of rumen microbiota in early weaned lambs. Microorganisms. 2022;10(1):144. 10.3390/microorganisms10010144. 35056593 10.3390/microorganisms10010144 PMC8779368

[147] Loch M, Dorbek-Sundström E, Husso A, Pessa-Morikawa T, Niine T, Kaart T, et al. Associations of neonatal dairy calf faecal microbiota with inflammatory markers and future performance. Animals (Basel). 2024;14(17):2533. 10.3390/ani14172533. 39272317 10.3390/ani14172533 PMC11394540

[148] Yan X, Si H, Zhu Y, Li S, Han Y, Liu H, et al. Integrated multi-omics of the gastrointestinal microbiome and ruminant host reveals metabolic adaptation underlying early life development. Microbiome. 2022;10:222. 10.1186/s40168-022-01396-8. 10.1186/s40168-022-01396-8 PMC9743514 36503572

[149] Zhong X, Zhang Z, Wang S, Cao L, Zhou L, Sun A, et al. Microbial-driven butyrate regulates jejunal homeostasis in piglets during the weaning stage. Front Microbiol. 2019;9:3335. 10.3389/fmicb.2018.03335. 30713531 10.3389/fmicb.2018.03335 PMC6345722

[150] Cui Z, Wu S, Liu S, Sun L, Feng Y, Cao Y, et al. From maternal grazing to barn feeding during the pre-weaning period: altered gastrointestinal microbiota contributes to change the development and function of the rumen and intestine of Yak calves. Front Microbiol. 2020;11:485. 10.3389/fmicb.2020.00485. 32308649 10.3389/fmicb.2020.00485 PMC7145940

[151] Jin C, Wu S, Liang Z, Zhang J, Lei X, Bai H, et al. Multi-omics reveal mechanisms of high enteral starch diet mediated colonic dysbiosis via microbiome-host interactions in young ruminant. Microbiome. 2024;12:38. 10.1186/s40168-024-01760-w. 10.1186/s40168-024-01760-w PMC10893732 38395946

[152] Kolathingal-thodika N, Elayadeth-meethal M, Dunshea FR, Eckard R, Flavel M, Chauhan SS. Is early life programming a promising strategy for methane mitigation and sustainable intensification in ruminants ? Sci Total Environ. 2025;982:179654. 10.1016/j.scitotenv.2025. 40359832 10.1016/j.scitotenv.2025.179654

[153] Jiao J, Wu J, Zhou C, Tang S, Wang M, Tan Z. Composition of ileal bacterial community in grazing goats varies across non-rumination, transition and rumination stages of life. Front Microbiol. 2016;7:1364. 10.3389/fmicb.2016.01364. 27656165 10.3389/fmicb.2016.01364 PMC5011132

[154] Suárez-Martínez C, Santaella-Pascual M, Yagüe-Guirao G, Martínez-Graciá C. Infant gut microbiota colonization: influence of prenatal and postnatal factors, focusing on diet. Front Microbiol. 2023;14:1236254. 10.3389/fmicb.2023. 37675422 10.3389/fmicb.2023.1236254 PMC10478010

[155] Klein-Jöbstl D, Quijada NM, Dzieciol M, Feldbacher B, Wagner M, Drillich M, et al. Microbiota of newborn calves and their mothers reveals possible transfer routes for newborn calves’ gastrointestinal microbiota. PLoS ONE. 2019;14(8):e0220554. 10.1371/journal.pone.0220554. 31369600 10.1371/journal.pone.0220554 PMC6675284

[156] Welch CB, Ryman VE, Pringle TD, Lourenco JM. Utilizing the gastrointestinal microbiota to modulate cattle health through the microbiome-gut-organ axes. Microorganisms. 2022; 10(7):1391. 10.3390/microorganisms10071391. 10.3390/microorganisms10071391 PMC9324549 35889109

[157] Wang J, Fan H, Li M, Zhao K, Xia S, Chen Y, et al. Integration of non-coding RNA and mRNA profiles reveals the mechanisms of rumen development induced by different types of diet in calves. Genes. 2023;14(5):1093. 10.3390/genes14051093. 37239453 10.3390/genes14051093 PMC10218261

[158] Thomson S, Hamilton CA, Hope JC, Katzer F, Mabbott NA, Morrison LJ, et al. Bovine cryptosporidiosis: impact, host-parasite interaction and control strategies. Vet Res. 2017;48:42. 10.1186/s13567-017-0447-0. 10.1186/s13567-017-0447-0 PMC5553596 28800747

[159] Lachnit T, Ulrich L, Willmer FM, Hasenbein T, Steiner LX, Wolters M, et al. Nutrition-induced changes in the microbiota can cause dysbiosis and disease development. mBio. 2025;16(4):e0384324. 10.1128/mbio.03843-24. 10.1128/mbio.03843-24 PMC11980362 39998180

[160] Gamsjäger L, Cirone KM, Schluessel S, Campsall M, Herik A, Lahiri P, et al. Host innate immune responses and microbiome profile of neonatal calves challenged with *Cryptosporidium parvum* and the effect of bovine colostrum supplementation. Front Cell Infect Microbiol. 2023;13:1165312. 10.3389/fcimb.2023.1165312. 37207189 10.3389/fcimb.2023.1165312 PMC10189047

[161] Luo T, Li Y, Zhang W, Liu J, Shi H. Rumen and fecal microbiota profiles associated with immunity of young and adult goats. Front Immunol. 2022;13:978402. 10.3389/fimmu.2022.978402. 36177023 10.3389/fimmu.2022.978402 PMC9513485

[162] Hares MF, Griffiths BE, Johnson F, Nelson C, Haldenby S, Stewart CJ, et al. Specific pathway abundances in the neonatal calf faecal microbiome are associated with susceptibility to *Cryptosporidium parvum* infection: a metagenomic analysis. Anim Microbiome. 2023;5:43. 10.1186/s42523-023-00265-5. 10.1186/s42523-023-00265-5 PMC10496319 37700351

[163] Shen Y, Li Y, Wu T, Dong Q, Deng Q, Liu L, et al. Early microbial intervention reshapes phenotypes of newborn Bos taurus through metabolic regulations. Gigascience. 2024; 13:giad118. 10.1093/gigascience/giad118. 10.1093/gigascience/giad118 PMC10787367 38217406

[164] Yáñez-Ruiz DR, Abecia L, Newbold CJ. Manipulating rumen microbiome and fermentation through interventions during early life: a review. Front Microbiol. 2015;6:1133. 10.3389/fmicb.2015.01133. 26528276 10.3389/fmicb.2015.01133 PMC4604304

[165] Bian G, Yu S, Cheng C, Huang H, Liu J. Ruminal microbiota-host crosstalks promote ruminal epithelial development in neonatal lambs with alfalfa hay introduction. mSystems. 2024; 9(2):e0103423. 10.1128/msystems.01034-23. 10.1128/msystems.01034-23 PMC10878101 38179946

[166] Malmuthuge N, Chen Y, Liang G, Widenmann A, Guan LL. Region-specific establishment of bacterial communities in the small intestine of neonatal calves from birth. Anim Nutr. 2024;1(1):1–34. 10.1017/anr.2024.4.

[167] Greenwood EC, Torok VA, Agenbag B, Hynd PI. Manipulation of neonatal ruminal populations at birth results in sustained effects on microbial populations and measures of health and production in merino and suffolk lambs. Livest Sci. 2024; 105406.10.1016/j.livsci.2024.105406.

[168] Wang Y, An M, Zhang Z, Zhang W, Kulyar MF e A, Iqbal M, et al. Effects of milk replacer-based Lactobacillus on growth and gut development of Yaks’ calves: a gut microbiome and metabolic study . Microbiol Spectr. 2022;10(4):e0115522. 10.1128/spectrum.01155-22. 10.1128/spectrum.01155-22 PMC9431445 35771011

[169] Badman J, Daly K, Kelly J, Moran AW, Cameron J, Watson I, et al. The effect of milk replacer composition on the intestinal microbiota of pre-ruminant dairy calves. Front Vet Sci. 2019;6:371. 10.3389/fvets.2019.00371. 31709269 10.3389/fvets.2019.00371 PMC6821647

[170] Wang Y, Xia H, Yang Q, Yang D, Liu S, Cui Z. Evaluating starter feeding on ruminal function in yak calves : combined 16S rRNA sequencing and metabolomics. Front Microbiol. 2022;13:821613. 10.3389/fmicb.2022.821613. 35733970 10.3389/fmicb.2022.821613 PMC9207444

[171] Yang B, Yang B, Le J, Wu P, Liu J, Guan LL, et al. Alfalfa intervention alters rumen microbial community development in Hu lambs during early life. Front Microbiol. 2018;9:574. 10.3389/fmicb.2018.00574. 29636743 10.3389/fmicb.2018.00574 PMC5881016

[172] Hays KE, Pfaffinger JM, Ryznar R. The interplay between gut microbiota, short- chain fatty acids, and implications for host health and disease. Gut Microbes. 2024;16(1):2393270. 10.1080/19490976.2024.2393270. 10.1080/19490976.2024.2393270 PMC11407412 39284033

[173] Abbas M, Abbas G, Hashmi AH, Jaffery S, Li Y, Zhao G, et al. Sustainable AGP alternatives : a systems approach to non-antibiotic growth regulators standardization, synergistic formulation and environmental safety. Front Vet Sci. 2026;12:1695160. 10.3389/fvets.2025.1695160. 10.3389/fvets.2025.1695160 PMC12903921 41695212

[174] Li A, Yang Y, Qin S, Lv S, Jin T, Li K, et al. Microbiome analysis reveals gut microbiota alteration of early-weaned Yimeng black goats with the effect of milk replacer and age. Microb Cell Fact. 2021;20:78. 10.1186/s12934-021-01568-5. 10.1186/s12934-021-01568-5 PMC8010993 33789672

[175] O’Hara E, Kelly A, McCabe MS, Kenny DA, Guan LL, Waters SM. Effect of a butyrate-fortified milk replacer on gastrointestinal microbiota and products of fermentation in artificially reared dairy calves at weaning. Sci Rep. 2018;8:14901. 10.1038/s41598-018-33122-6. 10.1038/s41598-018-33122-6 PMC6175921 30297834

[176] Zhuang Y, Guo W, Cui K, Tu Y, Diao Q, Zhang N, et al. Altered microbiota, antimicrobial resistance genes, and functional enzyme profiles in the rumen of yak calves fed with milk replacer. Microbiol Spectr. 2024;12(1):e0131423. 10.1128/spectrum.01314-23. 38014976 10.1128/spectrum.01314-23 PMC10871699

[177] Huang Y, Wang G, Zhang Q, Chen Z, Li C, Wang W, et al. Effects of milk replacer feeding level on growth performance, rumen development and the ruminal bacterial community in lambs. Front Microbiol. 2023;13:1069964. 10.3389/fmicb.2022. 36704552 10.3389/fmicb.2022.1069964 PMC9871810

[178] Zened A, Julien C, Cauquil L, Pascal G, Canlet C, Tremblay-Franco M, et al. Milk replacer feeding once or twice a day did not change the ruminal metabolomic profile and the microbial diversity of dairy calves from birth to weaning. J Dairy Sci. 2024;107(8):5574–86. 10.3168/jds.2023-24327. 38460877 10.3168/jds.2023-24327

[179] Wei X, Zou J, Zhang Y, Yang J, Wang J, Wang Y, et al. Effects of milk, milk replacer, and milk replacer plus ethoxyquin on the growth performance, weaning stress, and the fecal microbiota of Holstein dairy calves. Front Microbiol. 2023;14:1113518. 10.3389/fmicb.2023.1113518. 36992934 10.3389/fmicb.2023.1113518 PMC10040532

[180] Han Z, Li A, Pei L, Li K, Jin T, Li F, et al. Milk replacer supplementation ameliorates growth performance and rumen microbiota of early-weaning Yimeng black goats. Front Vet Sci. 2020;7:572064. 10.3389/fvets.2020.572064. 33240951 10.3389/fvets.2020.572064 PMC7669828

[181] Rodríguez GB, Carmona DAC, Elghandour MMY, Salem AZM, Soto HR, Sánchez RR, et al. Sustainable use of sodium butyrate as a source of bioactive additive: impact on calf growth performance, rumen fermentation characteristics, and microbial count. Biomass Conv Bioref. 2024;14(5):6229–35. 10.1007/s13399-022-02707-7.

[182] Olagunju LK, Casper DP, Officer M, Klanderman K, Anele UY. Male Holstein calves fed a milk replacer and pelleted calf starter containing a botanical extract or a direct-fed microbial alone or in combination. J Dairy Sci. 2024;107(12):10838–50. 10.3168/jds.2024-25137. 39265831 10.3168/jds.2024-25137

[183] Zhang K, Qian Q, Mao Y, Xu Y, Yang Y, Chen Y, et al. Characterization of growth phenotypes and gastrointestinal tract microbiota in sheep fed with caragana. J Appl Microbiol. 2021;131(6):2763–79. 10.1111/jam.15138. 33998744 10.1111/jam.15138

[184] Lin L, Trabi EB, Xie F, Mao S. Comparison of the fermentation and bacterial community in the colon of Hu sheep fed a low-grain, non-pelleted, or pelleted high-grain diet. Appl Microbiol Biotechnol. 2021;105(5):2071–80. 10.1007/s00253-021-11158-5. 33559720 10.1007/s00253-021-11158-5

[185] Azad MB, Konya T, Guttman DS, Field CJ, Sears MR, Hayglass KT, et al. Infant gut microbiota and food sensitization: associations in the first year of life. Clin Exp Allergy. 2015;45(3):632–43. 10.1111/cea.12487. 25599982 10.1111/cea.12487

[186] Ellison MJ, Conant GC, Cockrum RR, Austin KJ, Truong H, Becchi M, et al. Diet alters both the structure and taxonomy of the ovine gut microbial ecosystem. DNA Res. 2014;21(2):115–25. 10.1093/dnares/dst044. 24170804 10.1093/dnares/dst044 PMC3989484

[187] Guo H, Li B, Gao M, Li Q, Gao Y, Dong N, et al. Dietary nutritional level affects intestinal microbiota and health of goats. Microorganisms. 2022;10(12):2322. 10.3390/microorganisms10122322. 36557575 10.3390/microorganisms10122322 PMC9781347

[188] Quijada NM, Bodas R, Lorenzo JM, Schmitz-Esser S, Rodríguez-Lázaro D, Hernández M. Dietary supplementation with sugar beet fructooligosaccharides and garlic residues promotes growth of beneficial bacteria and increases weight gain in neonatal lambs. Biomolecules. 2020;10(8):1179. 10.3390/biom10081179. 32823755 10.3390/biom10081179 PMC7465112

[189] Fomenky BE, Do DN, Talbot G, Chiquette J, Bissonnette N, Chouinard YP, et al. Direct-fed microbial supplementation influences the bacteria community composition of the gastrointestinal tract of pre- and post-weaned calves. Sci Rep. 2018;8:14147. 10.1038/s41598-018-32375-5. 10.1038/s41598-018-32375-5 PMC6148029 30237565

[190] Du S, Bu Z, You S, Bao J, Jia Y. Diversity of growth performance and rumen microbiota vary with feed types. Front Sustain Food Syst. 2022;6:1004373. 10.3389/fsufs.2022.1004373Y.

[191] Cui Z, Meng Q, Ma W, Zhang X, Zhou Z, Ren L. Diversity of the intestinal bacteria of cattle fed on diets with different doses of gelatinized starch-urea. Indian J Microbiol. 2015;55(3):269–77. 10.1007/s12088-015-0526-8. 26063936 10.1007/s12088-015-0526-8 PMC4456495

[192] Song C, Zhang T, Xu D, Zhu M, Mei S, Zhou B, et al. Impact of feeding dried distillers’ grains with solubles diet on microbiome and metabolome of ruminal and cecal contents in Guanling yellow cattle. Front Microbiol. 2023;14:1171563. 10.3389/fmicb.2023.1171563. 37789852 10.3389/fmicb.2023.1171563 PMC10543695

[193] Zhang R, Zhong Z, Ma H, Lin L, Xie F, Mao S, et al. Mucosal microbiota and metabolome in the ileum of Hu sheep offered a low-grain, pelleted or non-pelleted high-grain diet. Front Microbiol. 2021;12:718884. 10.3389/fmicb.2021.718884. 34512596 10.3389/fmicb.2021.718884 PMC8427290

[194] Xu S, Wang S, Zhao W, Bi Y, Diao Q, Tu Y. Multi-omics reveals that forage fiber promotes rumen development of pre-weaning calves compared to non-forage fiber. Research Square. 2023. 10.21203/rs.3.rs-2498511/v1.

[195] Auffret MD, Dewhurst RJ, Duthie CA, Rooke JA, John Wallace R, Freeman TC, et al. The rumen microbiome as a reservoir of antimicrobial resistance and pathogenicity genes is directly affected by diet in beef cattle. Microbiome. 2017;5:159. 10.1186/s40168-017-0378-z. 10.1186/s40168-017-0378-z PMC5725880 29228991

[196] Wei Z, Xie X, Xue M, Valencak TG, Liu J, Sun H. The effects of non-fiber carbohydrate content and forage type on rumen microbiome of dairy cows. Animals (Basel). 2021;11(12):3519. 10.3390/ani11123519. 34944297 10.3390/ani11123519 PMC8698165

[197] Ribeiro CS, Granja-Salcedo YT, Messana JD, et al. Feeding increasing concentrate to Tifton 85 hay ratios modulated rumen fermentation and microbiota in Nellore feedlot steers. J Agric Sci. 2015;153(6):1116–27. 10.1017/S0021859615000337.

[198] Fu X, Zhang Y, Shi B, Wu X, Zhao H, Xin Z, et al. Benzoic acid metabolism and lipopolysaccharide synthesis of intestinal microbiome affects the health of ruminants under free-range and captive mode. Life Basel. 2022;12(7):1071. 10.3390/life12071071. 35888160 10.3390/life12071071 PMC9317595

[199] Federiconi A, Ghiaccio F, Mammi L, Cavallini D, Visentin G, Formigoni A, et al. Changes in the rumen microbial community composition of dairy cows subjected to an acidogenic diet. J Dairy Sci. 2024;107(10):7810–21. 10.3168/jds.2023-24599. 38825118 10.3168/jds.2023-24599

[200] Rico DE, Preston SH, Risser JM, Harvatine KJ. Rapid changes in key ruminal microbial populations during the induction of and recovery from diet-induced milk fat depression in dairy cows. Br J Nutr. 2015;114(3):358–67. 10.1017/S0007114515001865. 26123320 10.1017/S0007114515001865

[201] Dias J, Marcondes MI, Noronha MF, Resende RT, Machado FS, Mantovani HC, et al. Effect of pre-weaning diet on the ruminal archaeal, bacterial, and fungal communities of dairy calves. Front Microbiol. 2017;8:1553. 10.3389/fmicb.2017.01553. 28861065 10.3389/fmicb.2017.01553 PMC5559706

[202] Chai J, Lv X, Diao Q, Usdrowski H, Zhuang Y, Huang W, et al. Solid diet manipulates rumen epithelial microbiota and its interactions with host transcriptomic in young ruminants. Environ Microbiol. 2021;23(11):6557–68. 10.1111/1462-2920.15757. 34490978 10.1111/1462-2920.15757 PMC9292864

[203] Mao SY, Huo WJ, Zhu WY. Microbiome-metabolome analysis reveals unhealthy alterations in the composition and metabolism of ruminal microbiota with increasing dietary grain in a goat model. Environ Microbiol. 2016;18(2):525–41. 10.1111/1462-2920.12724. 25471302 10.1111/1462-2920.12724

[204] Hartinger T, Pacífico C, Sener-Aydemir A, Poier G, Kreuzer-Redmer S, Terler G, et al. Dietary carbohydrate sources differently prime the microbial ecosystem but not the epithelial gene expression profile along the complete gut of young calves. Anim Microbiome. 2024;6:12. 10.1186/s42523-024-00297-5. 10.1186/s42523-024-00297-5 PMC10935977 38481349

[205] Li Z, Mu C, Xu Y, Shen J, Zhu W. Changes in the solid, liquid, and epithelium-associated bacterial communities in the rumen of Hu lambs in response to dietary urea supplementation. Front Microbiol. 2020;11:244. 10.3389/fmicb.2020.00244. 32153533 10.3389/fmicb.2020.00244 PMC7046558

[206] Liang J, Ali S, Lv C, Yang H, Zhao X, Ni X, et al. Dietary protein levels modulate the gut microbiome composition through fecal samples derived from lactating ewes. Front Endocrinol (Lausanne). 2023;14:1194425. 10.3389/fendo.2023.1194425. 37621652 10.3389/fendo.2023.1194425 PMC10446493

[207] Yang CT, Si B-W, Diao QY, Jin H, Zeng SQ, Tu Y. Rumen fermentation and bacterial communities in weaned Chahaer lambs on diets with different protein levels. J Integr Agric. 2016;15(7):1564–74. 10.1016/S2095-3119(15)61217-5.

[208] Frutos J, Andres S, Yáñez-Ruiz DR, Benavides J, López S, Santos A, et al. Early feed restriction of lambs modifies ileal epimural microbiota and affects immunity parameters during the fattening period. Animal. 2018;12(10):2115–22. 10.1017/S1751731118000836. 29679995 10.1017/S1751731118000836

[209] Wolff SM, Ellison MJ, Hao Y, Cockrum RR, Austin KJ, Baraboo M, et al. Diet shifts provoke complex and variable changes in the metabolic networks of the ruminal microbiome. Microbiome. 2017;5:60. 10.1186/s40168-017-0274-6. 10.1186/s40168-017-0274-6 PMC5465553 28595639

[210] Du W, Wang X, Hu M, Hou J, Du Y, Si W, et al. Modulating gastrointestinal microbiota to alleviate diarrhea in calves. Front Microbiol. 2023;14:1181545. 10.3389/fmicb.2023.1181545. 10.3389/fmicb.2023.1181545 PMC10286795 37362944

[211] Gorka et al. Effect of probiotic and nucleotide supplementation in milk replacer on growth performance and fecal bacteria in calves. Livestock Sci. 2021;250. 10.1016/j.livsci.2021.104556.

[212] Idowu PA, Mbambalala L, Akinmoladun OF, Idowu AP. Gut microbiome modulation by probiotics : implications for livestock growth performance and health-narrative review. Appl Microbiol. 2025;5(4):149. 10.3390/applmicrobiol5040149.

[213] Al-Galiby MKA, Al-Hassnawi IA, Al-Hassnawi MM. Effect of different levels of probiotics on rumen environment and microbial condition in local goat kids. J Glob Innov Agric Sci. 2023;11(2):147–52. 10.22194/JGIAS/23.1076.

[214] Astuti WD, Ridwan R, Fidriyanto R, Rohmatussolihat R, Sari NF, Sarwono KA, et al. Changes in rumen fermentation and bacterial profiles after administering Lactiplantibacillus plantarum as a probiotic. Vet World. 2022;15(8):1969–74. 10.14202/vetworld.2022. 36313835 10.14202/vetworld.2022.1969-1974 PMC9615511

[215] Dar AH, Singh SK, Rahman JU, Ahmad SF. The effects of probiotic *Lactobacillus acidophilus* and/or prebiotic mannan oligosaccharides on growth performance, nutrient utilization blood metabolites, faecal bacteria, and economics of crossbred calves. Iran J Vet Res. 2022;23(4):322–30. 10.22099/IJVR.2022.. 36874183 10.22099/IJVR.2022.42992.6259 PMC9984140

[216] Devyatkin V, Mishurov A, Kolodina E. Probiotic effect of *Bacillus subtilis* B-2998D, B-3057D, and *Bacillus licheniformis* B-2999D complex on sheep and lambs. J Adv Vet Anim Res. 2021;8(1):146–57. 10.5455/javar.2021.h497. 33860025 10.5455/javar.2021.h497 PMC8043341

[217] Fernández-Ciganda S, Fraga M, Zunino P. Probiotic lactobacilli administration induces changes in the fecal microbiota of preweaned dairy calves. Probiotics Antimicrob Proteins. 2022;14(5):804–15. 10.1007/s12602-021-09834-z. 34390476 10.1007/s12602-021-09834-z

[218] Maake TW, Aiyegoro OA, Adeleke MA. Effects of *Lactobacillus rhamnosus* and *Enterococcus faecalis* supplementation as direct-fed microbials on rumen microbiota of Boer and Speckled goat breeds. Vet Sci. 2021;8(6):103. 10.3390/vetsci8060103. 34200410 10.3390/vetsci8060103 PMC8229190

[219] Mao H, Ji W, Yun Y, Zhang Y, Li Z, Wang C. Influence of probiotic supplementation on the growth performance, plasma variables, and ruminal bacterial community of growth-retarded lamb. Front Microbiol. 2023;14:1216534. 10.3389/fmicb.2023.1216534. 37577421 10.3389/fmicb.2023.1216534 PMC10413120

[220] Schofield BJ, Lachner N, Le OT, McNeill DM, Dart P, Ouwerkerk D, et al. Beneficial changes in rumen bacterial community profile in sheep and dairy calves as a result of feeding the probiotic *Bacillus amyloliquefaciens* H57. J Appl Microbiol. 2018;124(3):855–66. 10.1111/jam.13688. 29314469 10.1111/jam.13688

[221] Ruvalcaba-Gómez JM, Villaseñor-González F, Espinosa-Martínez MA, Gómez-Godínez LJ, Rojas-Anaya E, Villagrán Z, et al. Growth performance and fecal microbiota of dairy calves supplemented with autochthonous lactic acid bacteria as probiotics in Mexican Western family dairy farming. Animals. 2023;13(18):2841. 10.3390/ani13182841. 37760240 10.3390/ani13182841 PMC10525134

[222] Wu Y, Nie C, Luo R, Qi F, Bai X, Chen H, et al. Effects of multispecies probiotic on intestinal microbiota and mucosal barrier function of neonatal calves infected with *E. coli* K99. Front Microbiol. 2022;12:813245. 10.3389/fmicb.2021.813245. 35154038 10.3389/fmicb.2021.813245 PMC8826468

[223] Li Y, Li X, Nie C, Wu Y, Luo R, Chen C, et al. Effects of two strains of *Lactobacillus* isolated from the feces of calves after fecal microbiota transplantation on growth performance, immune capacity, and intestinal barrier function of weaned calves. Front Microbiol. 2023;14:1249628. 10.3389/fmicb.2023.1249628. 37727287 10.3389/fmicb.2023.1249628 PMC10505964

[224] Cai X, Yi P, Chen X, Wu J, Lan G, Li S, et al. Intake of compound probiotics accelerates the construction of immune function and gut microbiome in Holstein calves. Microbiol Spectr. 2024;12(6):e0190923. 10.1128/spectrum.01909-23. 38651859 10.1128/spectrum.01909-23 PMC11237676

[225] Seifdavati J. The effects of a medical plant mixture and a probiotic on performance, antioxidant activity and weaning age of newborn Holstein calves. Iran J Appl Anim Sci. 2016;6:285–91.

[226] Yüksek N, Çatalkaya E, Başbuğan Y, Yayan M. Effect of probiotic on total antioxidant (TAS) and total oxidant (TOS) in treatment of newborn calf diarrhea. Turkish J Vet Res. 2021;5(1):11–5.

[227] Ayyat MS, El-Nagar HA, Wafa WM, Abd El-Latif KM, Mahgoub S, Al-Sagheer AA. Comparable evaluation of nutritional benefits of *Lactobacillus plantarum* and *Bacillus toyonensis* probiotic supplementation on growth, feed utilization, health, and fecal microbiota in pre-weaning male calves. Animals. 2023;13(21):3422. 10.3390/ani13213422. 37958177 10.3390/ani13213422 PMC10649314

[228] Antanaitis R, Džermeikaitė K, Krištolaitytė J, Armonavičiūtė E, Arlauskaitė S, Girdauskaitė A, et al. Effects of *Bacillus subtilis* on growth performance, metabolic profile, and health status in dairy calves. Animals. 2024;14(17):2489. 10.3390/ani14172489. 39272274 10.3390/ani14172489 PMC11394282

[229] Nishihara K, Villot C, Cangiano L, Guan LL, Steele M. Bacteria colonization and gene expression related to immune function in colon mucosa is associated with growth in neonatal calves regardless of live yeast supplementation. J Anim Sci Biotechnol. 2024;15:76. 10.1186/s40104-024-01030-7. 10.1186/s40104-024-01030-7 PMC11151515 38835065

[230] Abbas T, Orma A-H, Ibrahim T, Abd El-Wahab A. Effects of dietary probiotic supplementation on growth, rumen development and selected blood metabolites of growing calves. Mansoura Vet Med J. 2021;4:4. 10.21608/mvmj.2021.47831.1012.

[231] Magalhães J, Cappellozza BI, dos Santos TC, Inoe F, Pessoa Araújo Júnior J, Kurissio JK, et al. Effects of supplementing direct-fed microbials on health and growth of preweaning Gyr × Holstein dairy calves. J Dairy Sci. 2024;107(8):6117–30. 10.3168/jds.2023-24434. 38608942 10.3168/jds.2023-24434

[232] Amanullah SM, Jahan R, Rahman MM, Samad MA, Hossain SMJ. Effects of feeding probiotic on daily gain, fecal characteristics and blood metabolites in calf. Bangladesh J Anim Sci. 2023;52(2):45–54. 10.3329/bjas.v52i2.67212.

[233] Rybachuk ZV, Shkromada OI, Predko AV, Dudchenko YA. Influence of probiotics “Immunobacterin-D” on biocenoses and development of the gastrointestinal tract of calves. Sci Messenger LNU Vet Med Biotechnol. 2020;22(98):22–7. 10.32718/nvlvet9804.

[234] Khademi AR, Hashemzadeh F, Khorvash M, Mahdavi AH, Pazoki A, Ghaffari MH. Use of exogenous fibrolytic enzymes and probiotic in finely ground starters to improve calf performance. Sci Rep. 2022;12:11942. 10.1038/s41598-022-16070-0. 10.1038/s41598-022-16070-0 PMC9279382 35831399

